# Distinct epigenetic features of tumor-reactive CD8+ T cells in colorectal cancer patients revealed by genome-wide DNA methylation analysis

**DOI:** 10.1186/s13059-019-1921-y

**Published:** 2019-12-31

**Authors:** Rui Yang, Sijin Cheng, Nan Luo, Ranran Gao, Kezhuo Yu, Boxi Kang, Li Wang, Qiming Zhang, Qiao Fang, Lei Zhang, Chen Li, Aibin He, Xueda Hu, Jirun Peng, Xianwen Ren, Zemin Zhang

**Affiliations:** 1 BIOPIC, Beijing Advanced Innovation Center for Genomics, and School of Life Sciences, Peking University, Beijing, China; 2 Department of Surgery, Beijing Shijitan Hospital, Capital Medical University, Beijing, China; 3 Department of Surgery, Peking University Ninth School of Clinical Medicine, Beijing, China; 4 Peking-Tsinghua Center for Life Sciences, Academy for Advanced Interdisciplinary Studies, Peking University, Beijing, China; 5 Institute of Molecular Medicine, Peking-Tsinghua Center for Life Sciences, Beijing Key Laboratory of Cardiometabolic Molecular Medicine, Peking University, Beijing, China

**Keywords:** Colorectal cancer, Tumor-reactive T cells, Bystanders, Transcriptome, Methylome, Transcription factor, T cell exhaustion

## Abstract

**Background:**

Tumor-reactive CD8+ tumor-infiltrating lymphocytes (TILs) represent a subtype of T cells that can recognize and destroy tumor specifically. Understanding the regulatory mechanism of tumor-reactive CD8+ T cells has important therapeutic implications. Yet the DNA methylation status of this T cell subtype has not been elucidated.

**Results:**

In this study, we segregate tumor-reactive and bystander CD8+ TILs, as well as naïve and effector memory CD8+ T cell subtypes as controls from colorectal cancer patients, to compare their transcriptome and methylome characteristics. Transcriptome profiling confirms previous conclusions that tumor-reactive TILs have an exhausted tissue-resident memory signature. Whole-genome methylation profiling identifies a distinct methylome pattern of tumor-reactive CD8+ T cells, with tumor-reactive markers *CD39* and *CD103* being specifically demethylated. In addition, dynamic changes are observed during the transition of naïve T cells into tumor-reactive CD8+ T cells. Transcription factor binding motif enrichment analysis identifies several immune-related transcription factors, including three exhaustion-related genes (*NR4A1*, *BATF*, and *EGR2*) and *VDR*, which potentially play an important regulatory role in tumor-reactive CD8+ T cells.

**Conclusion:**

Our study supports the involvement of DNA methylation in shaping tumor-reactive and bystander CD8+ TILs, and provides a valuable resource for the development of novel DNA methylation markers and future therapeutics.

## Background

Colorectal cancer (CRC) is one of the most common cancers globally. CRC incidence has traditionally been the highest in affluent western countries, but it is now increasing rapidly in other countries with economic development. CRC treatment usually involves surgical removal of the tumor followed by adjuvant chemotherapy. In recent years, various kinds of immunotherapies, such as checkpoint blockade immunotherapy, have been used to enhance the antitumor potential. However, the responses to these treatments vary among patients. Recent literatures supported the notion that not all tumor-infiltrating lymphocytes (TILs) are tumor reactive [1–4]. Rather, bystander cells exist, which recognize a wide range of epitopes unrelated to cancer [1–4]. For tumor immunotherapy, it is valuable to target those cells of which the T cell receptor (TCR) repertoire is intrinsically tumor reactive. Co-expression of CD39 (ENTPD1) and CD103 (ITGAE) identifies such a unique T cell population [1, 3]. These cells have a tissue-resident memory (RM) signature with high expression of exhaustion markers, such as *PDCD1* and *HAVCR2* (also known as *Tim-3*). Interestingly, these TILs also exhibited low expression of *CCR7*, *CD127*, and *CD28*, indicative of an effector memory (EM) phenotype [3, 5]. Understanding the molecular basis of memory CD8+ T cells is key to developing effective therapies against cancers. Further investigation is needed to better distinguish the molecular natures of T_EM_ and this tumor-reactive T cell subtype.

Gene expression patterns, a key determinant for a cellular feature, are believed to be controlled by epigenetic changes [6]. Decoding the epigenome specific to tumor-reactive T cells is a pivotal step toward understanding the activation and expansion of this T cell population in cancer. However, how they are regulated epigenetically has not been addressed thus far. DNA methylation, a covalent modification of the DNA molecule, is a stable and heritable form of epigenetic modifications which participates in establishing and maintaining chromatin structures and regulates gene transcription [7]. In general, DNA methylation is critical for establishing stable gene-silencing programs, by affecting the interactions of DNA with chromatin proteins and transcription factors [8, 9]. Many studies have highlighted the importance of DNA methylation in regulating complex gene expression programs underlying immune responses [10–12]. It is thus important to define how the identities of tumor-reactive CD8+ T cells and bystanders are shaped at methylation level, including particular genes and networks.

In this study, we sorted tumor-reactive and bystander CD8+ TILs from treatment-naïve primary CRC patients based on the expression of CD39 and CD103, and naïve and T_EM_ CD8+ T cells from peripheral blood based on the expression of CD45RO, CD45RA, and CCR7. Adapted smart-seq2 and whole-genome bisulfite sequencing (bisulfite-seq) were performed to characterize the transcriptomic features, DNA methylome programming, methylation dynamics, and key transcription factors (TFs) in each T cell subtype. Our study can help understand the underlying mechanisms leading to the specific expression patterns of tumor-reactive CD8+ T cells, thereby facilitating the development of new therapeutic strategies targeting these cells.

## Results

### Transcriptomic characteristics of five CD8+ T cell subtypes

**Fig. 1:**
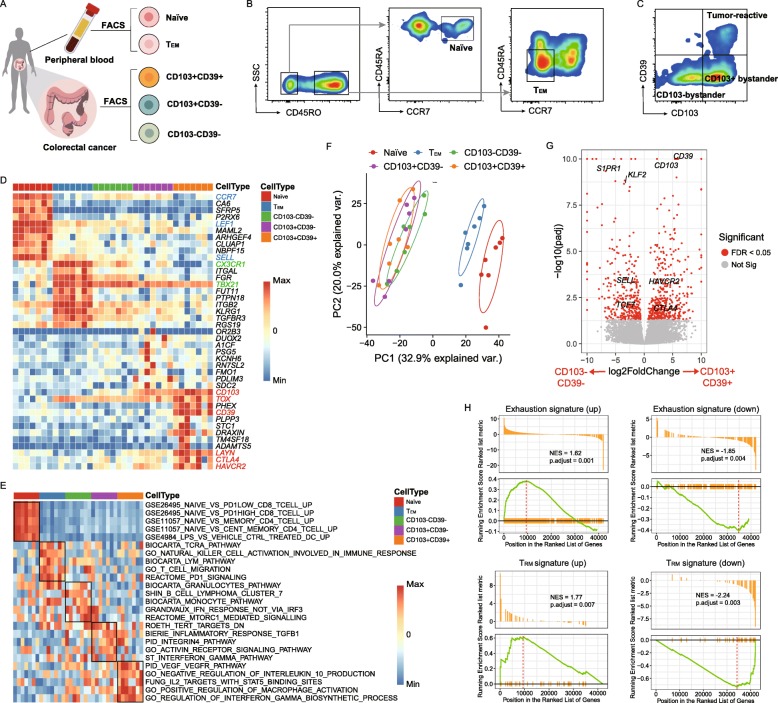
Comparative transcriptional analysis reveals tumor-reactive CD8+ T cells to have a T_RM_ signature with high expression of exhaustion markers. **a** Experimental design for the isolation of different CD8+ T cell populations from CRC patients. **b**, **c** Representative plots of FACS-isolated T cell populations. **d** Gene expression heat map of five CD8+ T cell populations. Rows represent signature genes, and columns represent cell types. Selective specifically expressed genes are marked in red. **e** GSVA was performed to identify enriched significant biological pathways in five CD8+ T cell subtypes. Five gene sets for each T cell population are depicted in a heat map. **f** PCA analysis of transcriptome expression of five CD8+ T cell populations. Each symbol represents one patient. **g** Volcano plot showing differential gene expression of CD103+CD39+ T cells vs. CD103−CD39− T cells (log2-transformed). Each red dot denotes an individual gene with a false-discovery rate (FDR) < 0.05. **h** Enrichment plot for the gene sets of “T cell exhaustion” and “T_RM_” in the transcriptome of CD103+CD39+ T cells vs. that of CD103−CD39− T cells by GSEA. NES, normalized enrichment score

Within CD8+ TILs, CD103+CD39+ T cells have been recently demonstrated to be tumor-reactive, while CD103−CD39− T cells and CD103+CD39− T cells are bystanders [1, 3]. To further characterize the transcriptional profiles of these cell populations, we isolated naïve, T_EM_, CD103+CD39+, CD103+CD39−, and CD103−CD39− T cell subtypes from eight CRC patients for gene expression profiling using adapted Smart-seq II (Fig. 1a–c; Additional file 1: Figure S1A, B; the “Methods” section). As shown in the heat map displaying differentially expressed genes (DEGs) among five CD8+ T cell subtypes, the naïve subtype exhibited high expression of known naïve markers *LEF1* and *SELL* (also known as *CD62L*) (Fig. 1d; Additional file 1: Figure S1C). T_EM_ subtype showed enhanced expression of classically defined T_EM_ molecules, such as *TBX21* [13] and *CX3CR1* [14] (Fig. 1d). Notably, CD103+CD39+ TILs displayed hallmarks of an “exhausted” phenotype, with high expression of *CTLA4*, *HAVCR2*, and *LAYN* (Fig. 1d; Additional file 1: Figure S1C, D). Recent literatures reported that the thymocyte selection-associated high mobility group box (TOX) protein is required for the development and maintenance of exhausted T cell populations in chronic infection [15–18]. Removal of its DNA binding domain reduced the expression of PD-1 and resulted in a more polyfunctional T cell phenotype [16]. Here, we observed that *TOX* expression is also upregulated (Fig. 1d; Additional file 1: Figure S2A). Intriguingly, our previous single-cell RNA-sequencing (scRNA-seq) data identified the specific expression of *TOX* in exhausted CD8+ TILs [19–21] (Additional file 1: Figure S2B-D). These data together supported the important role of *TOX* in intratumoral T cell exhaustion.

Gene set variation analysis (GSVA) showed that CD103+CD39+ subtype was enriched in biological processes associated with immunomodulation, such as “regulation of interferon gamma biosynthesis” and “negative regulation of IL10 production” [22, 23] (Fig. 1e). Furthermore, we analyzed effector function of these CD8 T cell subtypes by the expression of granzyme A/B/H, cytotoxic granules PRF1, interferon (IFN)-γ, and tumor necrosis factor (TNF). Interestingly, we found that exhausted CD103+CD39+ subtype still had relatively high expression of these cytotoxic proteins (Additional file 1: Figure S1C). Together with the GSVA results, it indicates that CD103+CD39+ subtype may not have lost their antitumor potential. Two-dimensional principal component analysis (PCA) revealed that naïve and T_EM_ subtypes were clearly grouped as distinct populations, whereas three CD8+ TIL subtypes appeared tightly clustered, indicative of a very similar transcriptional profile among these subtypes (Fig. 1f).

To gain a deeper understanding of tumor-reactive CD8+ T cells, we compared them with their counterpart, CD103−CD39− cells. CD103+CD39+ T cells highly expressed a set of 435 genes, including T cell exhaustion markers *CTLA4* and *HAVCR2* (Fig. 1g), but they exhibited lower expression of genes involved in T cell recirculation, such as *KLF2*, *SELL*, and *S1PR1* (Fig. 1g). Gene set enrichment analysis (GSEA) also revealed the presence of a molecular signature associated with T cell exhaustion and T_RM_ signatures (Fig. 1h). Then, we compared the transcriptome of CD103+CD39+ TILs with that of the T_EM_ subtype. Interestingly, CD103+CD39+ TILs also exhibited lower expression of *CCR7*, *CD127*, and *CD28*, as did T_EM_ subtype, consistent with previous findings (Additional file 1: Figure S1C) [3]. In addition, using a list of T_EM_ signature genes [5], we found most of these genes to exhibit low expression in naïve T cell subtype and high expression in other subtypes (Additional file 1: Figure S1E), further confirming the T_EM_ features in CD103+CD39+ T cells. Collectively, we again validated that tumor-reactive CD8+ T cells are in an exhausted state, characteristic of both T_RM_ and T_EM_ features.

### Global DNA methylation profiling across five CD8+ T cell subtypes

**Fig. 2:**
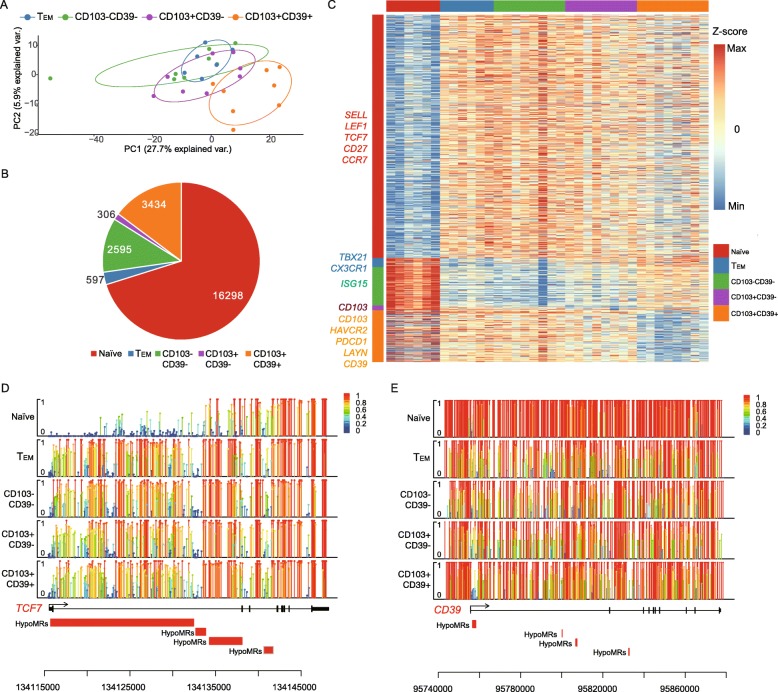
Whole-genome methylation profiling across multiple CD8+ T cell subtypes. **a** PCA analysis based on methylation profiles of CD8+ T cells in four T cell subtypes. **b** The graph shows the number of HypoMRs identified among five T cell subtypes. **c** Heat map showing the HypoMRs in the five subtypes. Color gradation from blue to red represents low to high DNA methylation levels. Selected genes associated with the HypoMRs were listed at the left side. **d**, **e** Lollipop plots for the nucleotide-resolution methylation level of the TCF7 and CD39 loci. Each covered cytosine is displayed as a bar with a large round head. The color and height of the bar indicate the methylation level

The phenotypic and functional changes that occur during CD8+ T cell differentiation are accompanied by genome-wide changes in DNA methylation programming. To comprehensively assess such methylation changes, we performed a genome-wide measurement of DNA methylation using bisulfite-seq (see the “Methods” section). For all samples, a median of ~ 26 million CpGs (45.1%) was covered (Additional file 1: Table S1). PCA analysis of the CpG methylation level of 5 kilobase (kb) genomic tiles among these T cell subtypes revealed that naïve CD8+ T cells were clearly grouped as a distinct population, whereas the rest were clustered (Fig. 2a; Additional file 1: Figure S3A). The T_EM_ subtype showed a similar methylation pattern to both bystander populations (Fig. 2a). Among the three TIL subtypes, CD103+CD39− T cells seem to possess a methylation signature that is intermediate between the CD103−CD39− and CD103+CD39+ T cells CD8+ T cells (Fig. 2a). Statistical analysis revealed that naïve subtype has the highest methylation level, which is line with its quiescent state (Additional file 1: Figure S3B).

To characterize the methylome further, we calculated hypomethylated regions (HypoMRs) for each T cell population in a “one vs. rest” fashion. We found a total of 23,230 HypoMRs in all CD8+ T subtypes (Fig. 2b). Of note, signature genes *LEF1*, *TCF7*, and *SELL* in naïve subtype and *TBX21* and *CX3CR1* in T_EM_ subtype were affected by specific HypoMRs, which corresponded to their enhanced expression (Fig. 2c; Additional file 1: Figure S4A, B). *ISG15*, a ubiquitin-like interferon-stimulated gene, was affected by a HypoMR in the CD103−CD39− subtype (Fig. 2c; Additional file 1: Figure S5). Its role as a central player in the host antiviral response might make it key to the immune functions of bystanders [24]. Notably, CD103+CD39+ T cells exhibited specific HypoMRs that affected the markers for tumor reactivity, *CD39* and *CD103* (Fig. 2c, e; Additional file 1: Figure S5). In addition, they also acquired an exhaustion-associated methylation program, with HypoMRs that affected the exhaustion markers *PDCD1*, *HAVCR2*, and *LAYN* (Fig. 2c; Additional file 1: Figure S5). Our methylation data suggested that the cell features observed in different CD8+ T cell subtypes may be shaped by altered DNA methylation profiles.

### The methylation dynamics of immune-related genes

**Fig. 3:**
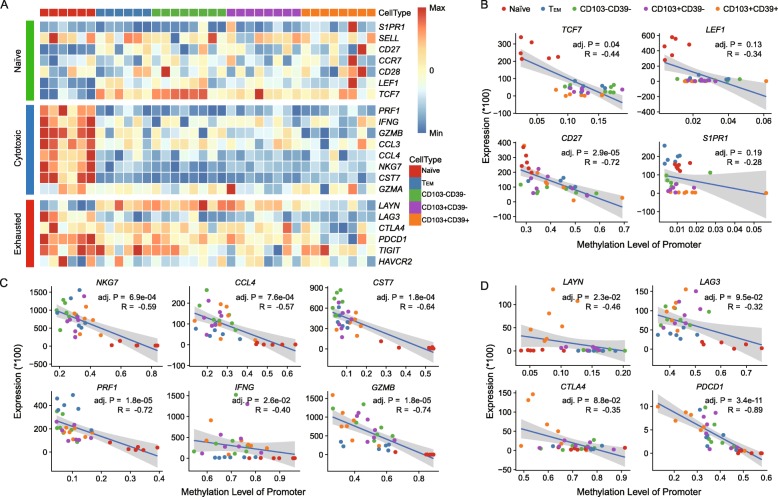
Immune function annotations. **a** Heat map showing methylation levels of selected T cell function-associated genes for each CD8+ T cell subtypes. **b**–**d** Scatter plots and trend lines were plotted to illustrate the correlation between the differences in mRNA expression and DNA methylation of promoters in three groups of immune signature genes for **b** naïve, **c** cytotoxic, and **d** exhausted T cells. R, Spearman’s correlation; adj. P, Benjamini-Hochberg adjusted *p* value

To understand the dynamic changes of methylation during the development of tumor-reactive CD8+ T cells, we analyzed promoter methylation levels of three immune signature gene sets for naïve, cytotoxic, and exhausted T cells (Fig. 3a; Additional file 1: Figure S6). We found that most signature genes for naïve T cells were demethylated in naïve subtypes, and displayed a higher level of methylation in other later T cell subtypes (Fig. 3a; Additional file 1: Figure S6). Of note, *TCF7* showed the most drastic changes (Fig. 3a; Additional file 1: Figure S6). In contrast, signature genes for cytotoxic T cells, including *PRF1*, *IFNG*, *GZMB*, *CCL3*, *CCL4*, *NKG7*, and *CST7*, were highly methylated in the naïve subtype and then became demethylated during naïve to T_EM_ differentiation (Fig. 3a; Additional file 1: Figure S6), indicating that a hypomethylation programming was acquired following the activation of naïve T cells. The hypomethylation statuses of cytotoxic signature genes were maintained within both bystander and tumor-reactive CD8+ T cells (Fig. 3a; Additional file 1: Figure S6). Finally, for exhausted signature genes, two inhibitory receptors *PDCD1* and *CTLA4* were found to be specifically demethylated within tumor-reactive CD8+ T cells (Fig. 3a; Additional file 1: Figure S6). Another inhibitory receptor *LAG3* was initially methylated in naïve cells, and became demethylated in later stages of T cell subtypes (Fig. 3a; Additional file 1: Figure S6). *LAYN*, which was reported as a novel exhausted marker in our previous study [20], underwent striking methylation during naïve to T_EM_ differentiation, maintained this methylation status in both bystander subtypes, and acquired demethylation in tumor-reactive T cells (Fig. 3a; Additional file 1: Figure S6).

Furthermore, to link DNA methylation states to gene expression, we performed correlation analyses of promoter methylation level and gene expression level. In most cases, the gene expression was found to be negatively correlated with their differential methylation status (Fig. 3b–d). For example, the expression level of *PDCD1* showed significant negative correlation with the methylation level of its promoter region (*p* = 5.7e−12). The similar methylation levels of *S1PR1* and *SELL* across different subtypes suggested that DNA methylation has a minor influence on their expression, and other factors may be responsible for their differential expression. In summary, the DNA methylation levels of immune-related genes were in dynamic changes from naïve T cells to tumor-reactive CD8+ T cells, resulting in divergent expression programs in each CD8+ T cell subtype.

### Key transcription factors for each CD8+ T cell subtype

**Fig. 4:**
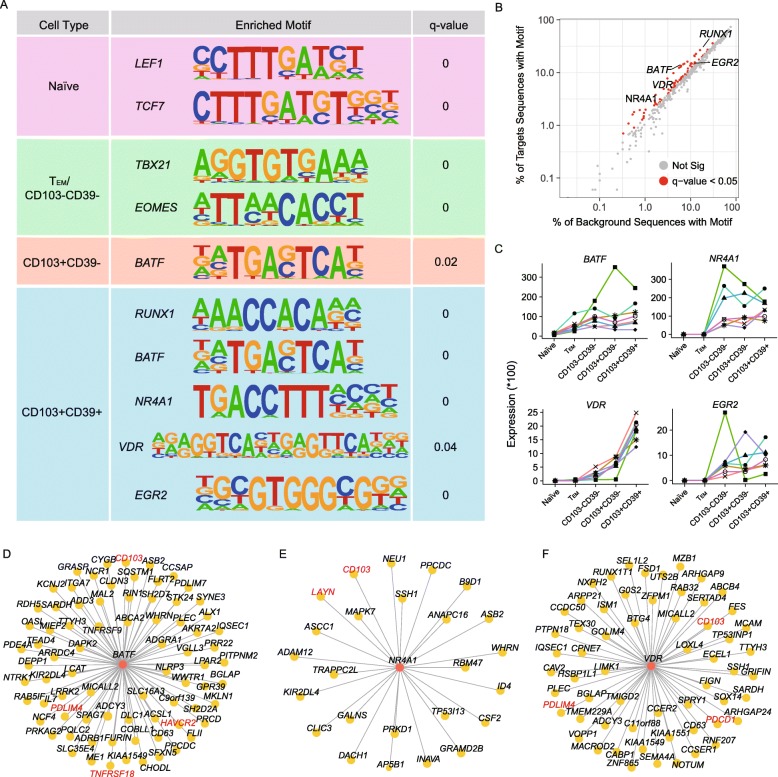
Key transcription factors and their targets for each CD8+ T cell subtype. **a** Selected TF motifs enriched in HypoMRs for five subtypes. *Q* value here represents adjusted *p* value reported by HOMER. **b** Target (*y*-axis) vs. background (*x*-axis) sequences with motif in CD103+CD39+ subtypes. **c** RNA expression of *BATF*, *NR4A1*, *VDR*, and *EGR2* in five subtypes. **d**–**f** Computed gene regulatory networks showing transcription factor *BATF* (**d**), *NR4A1* (**e**), and *VDR* (**f**) and their target genes. TFs and their targets of interest are labeled in red

Transcription factors (TFs) have a broad effect on cell state. To gain insight into the key regulators in each CD8+ T cell subtype, we performed TF binding motif enrichment analysis in HypoMRs. Those enriched TFs might have decisive roles in shaping the molecular features for each CD8+ T cell subtype. We found an overrepresentation of binding sites of *LEF1* and *TCF7* in naïve subtype (Fig. 4a), consistent with their key regulatory functions in naïve T cells [25, 26]. In both T_EM_ and CD103−CD39− T cells, the binding motifs of two T-box TFs *TBX21* and *EOMES*, which have been recognized as the master regulators of CD8+ T cell differentiation and function [13, 27], were found to be enriched within HypoMRs (Fig. 4a). Additionally, *BATF*, a transcription factor in the *AP-1* family, was found to be enriched within both CD103+CD39− and CD103+CD39+ TILs. *BATF* has been reported to initiate CD8+ T cell effector differentiation [28, 29]. This is consistent with the notion that increased expression of *BATF* in exhausted CD8+ T cells suppresses their effector function [29, 30].

Particularly, this analysis yielded 85 TF binding motifs that were significantly enriched in CD103+CD39+ T cells. In addition to *BATF*, significantly overrepresented motifs included *RUNX1*, *NR4A1* (also known as *NUR77*), vitamin D receptor (*VDR*), and *EGR2* (Fig. 4a, b), suggesting that these cells are under the control of a complex network of transcription factors. Of these five TFs, *BATF*, *NR4A1*, and *EGR2* have been reported to be associated with T cell exhaustion. *BATF* and *NR4A1* could regulate PD-1 to inhibit T cell function [30, 31]; *EGR2* targets *LAG3* and *4-1BB* regulate T cell dysfunction within tumors [32]. The other two TFs, *RUNX1* and *VDR*, have both been reported to be associated with T cell development [33, 34]. Interestingly, *RUNX1* mediates site-specific DNA demethylation at binding site in hematopoietic cells [35], which might explain the demethylated status here. Based on our RNA-Seq data, *BATF*, *NR4A1*, *VDR*, and *EGR2* all showed high expression in CD103+CD39+ subtype (Fig. 4c). Notably, the expression level of VDR showed progressive increase during T cell differentiation from naïve to tumor-reactive subtype. In contrast, we observed comparable expression of *RUNX1* among five subtypes (Additional file 1: Figure S7A).

Next, to understand the transcriptional regulatory networks, we used TF binding motifs to predict targets regulated by TFs in tumor-reactive CD8+ T cells (see the “Methods” section). Of note, exhaustion markers *LAYN*, *HAVCR2*, and *PDCD1* and tissue-resident marker *CD103* are predicted as the targets of these TFs (Fig. 4d–f; Additional file 1: Figure S7B, C). For example, *PDCD1* was predicted to be regulated by *VDR*, which has not been previously linked to T cell exhaustion. The regulatory role of *VDR* in *PCDC1* regulation and T cell exhaustion thus deserves further interrogation. Other predicted targets include T cell trafficking molecule *PDLIM4* and *TNFRSF18* (*GITR*) which is involved in regulating T cell programmed cell death (Fig. 4d, f; Additional file 1: Figure S7C) [36]. Collectively, these data support that tumor-reactive CD8+ T cells were regulated by a complex network of transcription factors.

### Transcription factors in regulating T cell exhaustion

**Fig. 5:**
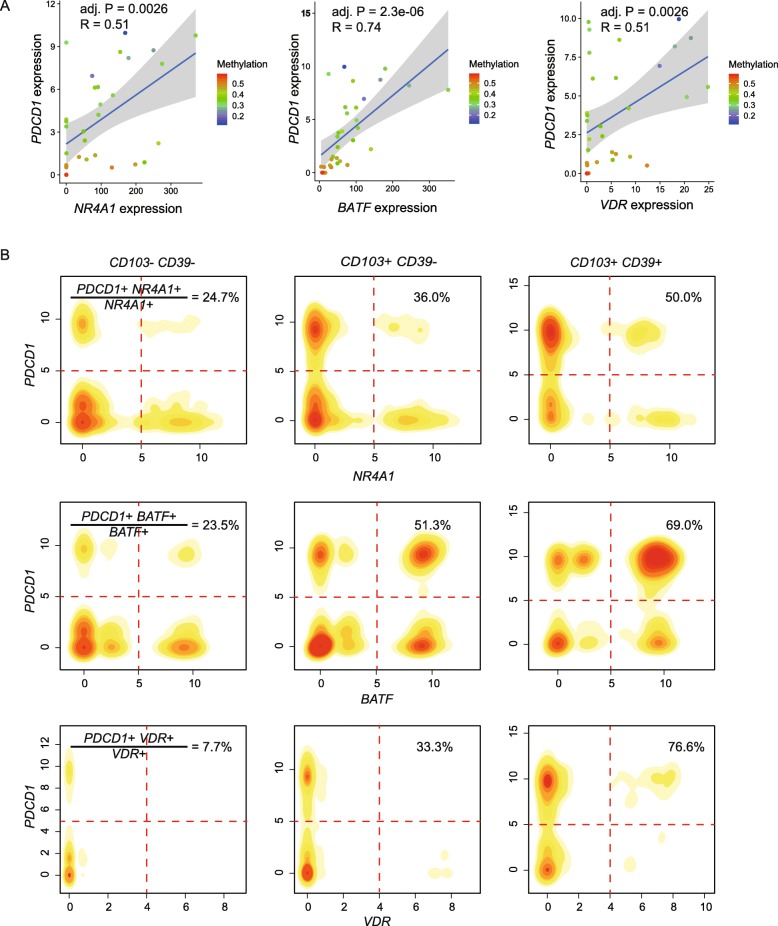
Effects of *NR4A1*, *BATF*, and *VDR* expression and methylation status of their PDCD1 binding sites on *PDCD1* expression. **a** Scatter plots and trend lines were plotted to illustrate the correlation between *PDCD1* expression and three TF (*NR4A1*, *BATF*, and *VDR*) expressions. Colors of dots represent the methylation levels of the promoter regions of *PDCD1*. Each dot represents a replicate of one patient. R, regression coefficient; adj. P, Benjamini-Hochberg adjusted *p* value. **b** In silico FACS analysis of our previous scRNA-seq data of CD8+ T cells from CRC patients. The percentages of double-positive cells for *PDCD1* and TF in the corresponding TF-positive cells are calculated in three TIL subtypes, respectively

Several predicted TFs in tumor-reactive cells have been reported to be associated with T cell exhaustion, such as *NR4A1* and *BATF* [30, 31]. In our study, a novel transcription factor *VDR* was identified to be upregulated within tumor-reactive cells. Since *PDCD1* upregulation is a hallmark of CD8+ T cell exhaustion, we here show an example of *PDCD1* regulated by three TFs to further discuss their relationship with T cell exhaustion. The recruitment of *NR4A1* by *PDCD1* was supported by ChIP-seq in a recent study [31]. Overall, a significant positive correlation existed between *PDCD1* expression and expression of *NR4A1*, *BATF*, and *VDR* in our own data (Fig. 5a). Particularly, when taking the promoter methylation level of *PDCD1* into account, several low expressing *PDCD1* cells with high *NR4A1* and *VDR* expression were found to have high DNA methylation levels of *PDCD1* promoters (Fig. 5a), suggesting the dominant role of DNA methylation in regulation *PDCD1* expression in these samples.

We used our previous scRNA-seq data of CD8+ T cells from CRC patients to further investigate the correlation of *PDCD1* and three TFs [19]. In silico FACS analysis was used to predict the percentages of double-positive cells for *PDCD1* and TF in the corresponding TF-positive cells among three TIL subtypes, respectively. Of note, the highest proportions were observed in CD103+CD39+ subtype for all three TFs (Fig. 5b), which further supported the positive regulation of *PDCD1* by three TFs. Overall, our study indicated the transcription regulation of *PDCD1* in CD103+CD39+ subtype by both TF expression and DNA methylation state of TF binding site, and suggested the contribution of these TFs in regulating T cell exhaustion.

Next, we analyzed the correlation of these three TFs with *TOX*, the novel T cell exhaustion marker. A strong positive correlation of *TOX* expression and *VDR* expression was observed, which further supports a role of *VDR* in regulating T cell exhaustion (Additional file 1: Figure S8). Future investigation should be considered that utilizes ChIP-seq to validate the exhaustion-associated targets of these TFs in tumor-reactive T cells.

## Discussion

Intratumoral CD8+ T cells are classified as tumor-reactive and bystanders based on their antigen specificities. Within CD8+ TILs, tumor-reactive T cells are enriched in CD103+CD39+ cells, while bystanders are abundant in CD39− cells [1, 3]. In eight patients, the proportions of tumor-reactive T cells ranged from 9.0 to 64.8%, with an average of 29.3% (Additional file 1: Figure S1B). A recent literature reported the intrinsic tumor-recognition potential of T cells in different human cancers by TCR profiling, and showed that tumor reactivity of TCRs was restricted to a minority of cells [2]. The relatively high proportion of tumor-reactive CD8+ T cells in our study suggested a certain number of bystander T cells were present. To further enrich tumor-reactive CD8+ population in the future, new markers need to be identified in addition to CD39 and CD103. In addition, it is intriguing to find out the molecular mechanism determining the various proportions of tumor-reactive T cells in different patients, which can have important clinical meanings. For instance, patients whose tumors had a higher percentage of tumor-reactive CD8 TILs at the time of surgery correlated with better overall survival [3]. Duhen et al. showed that the highest percentage of tumor-reactive CD8 TILs were found in melanoma and microsatellite instability (MSI)^high^ colon cancer, both tumors with high mutational burden [3]. Other molecular mechanism such as epigenetic regulation associated with proportions of tumor-reactive T cells remains to be explored in the future.

The exhausted state of tumor-reactive population impedes its therapeutic use. Reversing T cell exhaustion can reinvigorate immunity. However, a majority of patients lack durable response to immunotherapy such as immune checkpoint blockade, which is explained at least in part by the stable dysfunctional state of T cells shaped epigenetically [37, 38]. We added another layer of epigenetic regulation to intratumoral T cell exhaustion. Our in-depth methylation profiling identified the specific demethylation status of exhaustion markers, including *PDCD1*, *HAVCR2*, and *LAYN*. In addition, TF binding site analysis in HypoMRs for the enrichment of known TFs identified exhaustion-related TFs such as *NR4A1*, *BATF*, and *EGR2*. The current common epigenetic approaches for cancer treatment are the administration of demethylating agents such as azacitidine, which have a broad but undefined effect on the genome [39]. Advances in techniques for manipulating DNA methylation status in a targeted manner are anticipated to have significant clinical values [40–43]. Future therapeutic strategies of checkpoint blockade combined with epigenetic modifiers should be put into a brighter spotlight in the future.

We yielded putative biomarkers in mediating T cell exhaustion. Targeting these molecules might potentially augment T cell effector functions. Indeed, knockdown of *BATF* using shRNA-mediated gene-silencing enhanced T cell function [30]. *NR4A1* deletion enhanced immunity against tumor and chronic virus [31]. Intriguingly, *NR4A11*-deficient CD8+ T cells have lower *PDCD1* and *HAVCR2* expression [31]. In future study, roles of *VDR* and *RUNX1* in mediating T cells exhaustion need to be further defined and may provide new opportunities to reverse CD8+ T cell exhaustion.

## Conclusion

Methylation programming plays important roles in regulating gene expression. Herein, we showed that the transcriptomic features of tumor-reactive T cells were shaped by their distinct methylome profile. Intriguingly, tumor-reactive markers (*CD39* and *CD103*) and exhaustion markers (*PDCD1*, *LAYN*, and *HAVCR2*) were specifically demethylated. Integrated transcriptome and methylome profiling identified possible key regulators in the tumor-reactive subtype, including exhaustion-related TFs such as *NR4A1*, *BATF*, and *EGR2*. Novel TFs *RUNX1* and *VDR* identified here need further validation and may serve as potential therapeutic targets. Our understanding of transcriptome and methylome networks has important implications for the activation and expansion of tumor-reactive T cells, which will benefit future adoptive therapy.

## Methods

### Human specimen collection

This study was approved by the Research and Ethical Committee of Beijing Shijitan Hospital and complied with all relevant ethical regulations. Written informed consent was provided by all patients. Eight patients with CRC, including five women and three men, were enrolled and pathologically diagnosed with CRC at Beijing Shijitan Hospital. Fresh tumor and adjacent normal tissue samples (at least 2 cm from matched tumor tissues) were surgically resected from the above-described patients. None of them was treated with chemotherapy or radiation before tumor resection. The stages of these patients were classified according to the guidance of AJCC version 8. The available clinical characteristics are summarized in Additional file 1: Figure S1A.

### Single cell collection

Peripheral blood mononuclear cells (PBMCs) were isolated using HISTOPAQUE-1077 (Sigma-Aldrich) solution according to the manufacturer’s instructions. Briefly, 3 mL of fresh peripheral blood was collected prior to surgery in EDTA anticoagulant tubes and subsequently layered onto HISTOPAQUE-1077. After centrifugation, lymphocyte cells remained at the plasma-HISTOPAQUE-1077 interface and were carefully transferred to a new tube and washed twice with 1× PBS (Invitrogen). These lymphocytes were re-suspended with FACS buffer (PBS supplemented with 1% fetal bovine serum (FBS, Invitrogen)).

Tumors and adjacent normal tissues were cut into approximately 1 mm^3^ pieces in the RPMI-1640 medium (Invitrogen), and mechanically dissociated and enzymatically digested with MACS Tumor Dissociation Kit (Miltenyi Biotec) for 30 min on a rotor at 37 °C, according to the manufacturer’s instruction. The dissociated cells were subsequently passed through a 70-μm cell-strainer (BD) and centrifuged at 400*g* for 10 min. The cell pellets were suspended in red blood cell lysis buffer (Solarbio) and incubated on ice for 2 min to lyse red blood cells. After washing twice with PBS (Invitrogen), the cell pellets were re-suspended in FACS buffer.

### Antibodies and flow cytometry

The following fluorescent-labeled antibodies were used: BV711 anti-CD3 (BC96; 1:100—#300450), APC anti-CD8 (RPA-T8; 1:100—#301048), BV421 anti-CD45RA (HI100; 1:100—#47-0458-42), BV421 anti-CD45RO (HI100; 1:100—#47-0458-42), BV421 anti-CCR7 (HI100; 1:100—#47-0458-42), PE anti-CD39 (eBioA1; 1:100—#17-0399-42), and FITC anti-CD103 (B-Ly7 and Ber-ACT8; 1:100—#12-1038-42) (all from eBioscience). Cell surface staining was performed in FACS buffer. Stained cells were acquired on the FACS AriaII (all BD Biosciences) for cell sorting. Data were analyzed with FlowJo software (Treestar).

### Cell sorting, reverse transcription, amplification, and sequencing

One thousand cells of different subtypes including naïve and T_EM_ CD8+ T cells from PBMC, tumor-reactive CD8+ T cells, and two clusters of tumor bystander CD8+ T cells were enriched by gating 7AAD−CD3+CD8+CD45RO−CD45RA+CCR7+, 7AAD−CD3+CD8+CD45RO+CD45RA−CCR7−, 7AAD−CD3+CD8+CD103+CD39+, 7AAD−CD3+CD8+CD103+CD39−, and 7AAD−CD3+CD8+CD103−CD39−, respectively, and sorted into 0.2 mL tubes (Axygen) chilled to 4 °C, prepared with lysis buffer with 1 μL 10 mM dNTP mix (Invitrogen), 1 μL 10 μM Oligo dT primer, 1.9 μL 1% Triton X-100 (Sigma), and 0.1 μL 40 U μL^−1^ RNase Inhibitor (Takara).

Transcriptome amplifications were performed according to Smart-Seq2 protocol [44] with modification of reagent amount and PCR cycle numbers. The amplified cDNA products were purified with Agencourt XP DNA beads (Beckman), and the concentration of each sample was quantified by Qubit HsDNA kits (Invitrogen). Libraries were constructed and amplified using the TruePrep DNA Library Prep Kit V2 for Illumina (Vazyme Biotech). The libraries were then purified with Agencourt XP DNA beads and analyzed by fragment analyzer for quality assessment. Purified libraries were analyzed by an Illumina Hiseq 4000 sequencer with 150-bp pair-end reads.

### Whole-genome bisulfite-seq

Whole-genome bisulfite-seq was performed according to a previously published protocol [45]. Briefly, 1000 cells were sorted into lysis buffer by FACS; DNA was released after proteinase treatment at 50 °C and then subjected to bisulfite conversion. After bead-based purification, DNA was complemented with the biotinylated random primer Bio-P5-N9 (5′-biotin-CTACACGACGCTCTTCCGATCTNNNNNNNNN-3′) and 100 units of Klenow polymerase (3′ to 5′ exo-, New England BioLabs). This random priming was repeated seven times in total. Second strands were synthesized using another random primer, P7-N9 (5′-AGACGTGTGCTCTTCCGATCTNNNNNNNNN-3′), and final libraries were generated after 7 to 9 cycles of PCR amplification with the Illumina universal PCR primer and Illumina indexed primer.

### RNA-seq analysis

RNA-seq data were first processed to filter out low-quality reads with (1) “N” bases accounting for 3% read length, or (2) bases with quality < 3 account for 50% read length, or (3) containing adapter sequences. Then, kallisto [46] was used to quantify the abundances of transcripts. To summarize transcript-level abundance estimates for gene-level analysis, tximport [47] package from R bioconductor was used with parameter “countsFromAbundance = lengthScaledTPM” to correct library size and average transcript length across samples. Differential gene expression analysis was performed by using DESeq2 [48] package from R Bioconductor. Only the genes with adjusted *p* values less than 0.05 were considered to be differentially expressed. Normalized counts from DESeq2 were used to visualize the expression of genes and the downstream analysis. PCA analysis was performed with the R prcomp function on log2 (normalized counts + 1) expression values with specific gene subtypes, including highly variable genes across the five T cell populations (identified by R function *FindVariableFeatures* from Seurat [49] package) and significantly differential expression genes between CD103+CD39+ and CD103−CD39− samples. GSVA analysis was performed on log2 (normalized counts + 1) expression values by using GSVA [50] R package. To test the functional enrichment of CD103+CD39+ TILs vs. CD103−CD39− TILs, genes were ranked by fold-change difference, then using *GSEA* function from clusterProfiler [51] R package to test enrichment of T_RM_ and T_EX_ signatures, collected from previously published paper [3].

Naïve, cytotoxic, and exhausted scores were defined as the average expression of specific markers, with the expression level of each gene measured in log10-space. Seven markers for naïve T cells (*CCR7*, *LEF1*, *SELL*, *TCF7*, *S1PR1*, *CD27*, and *CD28*) were used for the naïve score. Eight markers for cytotoxic T cells (*NKG7*, *CCL4*, *CST7*, *PRF1*, *GZMA*, *GZMB*, *IFNG*, *CCL3*) and six markers for exhausted T cells (*TIGIT*, *HAVCR2*, *CTLA4*, *PDCD1*, *LAG3*, *LAYN*) were used to define the cytotoxic score and exhausted score. After delineating the naïve, cytotoxic, and exhausted scores of each T population, the Wilcoxon test was applied to compare the difference between each group, with *p* value < 0.05 considered significant.

### Bisulfite-seq analysis

Sequencing reads were trimmed of 9 bases for random primer sequences and removed the low-quality and adapter contaminated reads using trim galore (http://www.bioinformatics.babraham.ac.uk/projects/trim_galore). The cleaned reads were then mapped to the computationally bisulfite-converted human reference genome (GRCh38) by using Bismark [52] with paired-end mode (parameter settings: “bismark --pbat -N 1 -L 32”). Then, the unmapped reads after paired-end mapping were re-aligned to the same reference in single-end mode. Potential PCR duplicates were removed using *duplicate-remover* in MethPipe [53]. The lambda genome was added into human reference genome as extra chromosome to estimate the bisulfite conversion rate. We used *bsrate* from MethPipe to estimate bisulfite conversion rate, and samples with > 99% (or almost 99%) bisulfite conversion rates were retained for the DNA methylation analysis. Most post-alignment analysis was performed by functions from MethPipe [53] Package.

Methylation levels for each symmetric CpG site were calculated by the *methcounts* and *symmetric-cpgs* commands in MethPipe. Average CpG methylation levels of about 5 kb tils for all chromosomes in human genome were calculated with MethPipe *roimethstat* command for each T cell population. Only high-confidence genomic regions with at least 40 CpG observations from reads in the genomic tile were used for the PCA analysis (R prcomp function) to compare the overall methylation level in genome wide of the five T cell populations.

Specific hypomethylated regions (HypoMRs) for each T cell population were calculated in a “one vs. rest” fashion by using *radmeth regression*, *radmeth adjust*, and *radmeth merge* commands in MethPipe. Then, we overlapped these HypoMRs with promoter regions (defined as − 2.5 kb and 1 kb from transcription start site (RefSeq gene model downloaded from UCSC Table browser [54])) to identify genes affected by HypoMRs in each T cell population. The average methylation level of all HypoMRs in each T cell population was calculated by *roimethstat* command. To visualize the methylation pattern of given genes in different populations, lollipop plot from CGmapTools [55] was used. The Wilcoxon test was applied to compare the methylation levels of HypoMR between each group, with *p* value < 0.05 considered significant.

### Correlation of promoter methylation and RNA expression of immune-related genes

Here, we focused on three immune signature gene sets for naïve (*CCR7*, *LEF1*, *SELL*, *TCF7*, *S1PR1*, *CD27*, and *CD28*), cytotoxic (*NKG7*, *CCL4*, *CST7*, *PRF1*, *GZMA*, *GZMB*, *IFNG*, and *CCL3*) and exhausted (*TIGIT*, *HAVCR2*, *CTLA4*, *PDCD1*, *LAG3*, and *LAYN*) T cells. First, the average methylation levels of those gene promoter regions were calculated by using *roimethstat* command in each sample. RNA expression level of those genes was calculated by using the normalized counts from DESeq2. Spearman’s correlation was used to calculate the relationship of RNA expression level and promoter methylation level. *p* values were adjusted by the Benjamini-Hochberg method.

### Inferred regulation network construction

First, the enriched transcription factor motifs within HypoMRs were performed by HOMER [56] to identify possible key transcription factors in each T cell population. Furthermore, to investigate the possible target genes regulated by transcription factors in tumor-reactive T cells, transcription factor binding motifs were scanned through the promoter regions in the whole human genome (using *scanMotifGenomeWide.pl* in homer) to identify all possible target sites genome wide, and then, all the predicted target sites were intersected with the specific hypomethylated regions in tumor-reactive T cell population to construct the possible regulation network in each T cell population.

### In silico FACS for scRNA-seq data

scRNA-Seq was downloaded from GSE108989 [19]. A total of 1646 CD8+ T cells from tumor site were retained for our analysis, with the expression level of each gene measured by log2 (TPM + 1). Using cutoff 4 for the expression of gene CD103 and CD19, 25.9% cells were classified as CD103+CD39+, 41.0% were CD103+CD39−, and 29.9% were CD103−CD39−. Then, we used the scRNA-Seq data to investigate the co-expression of *PDCD1* and three TFs (*NR4A1*, *BATF*, and *VDR*). The percentages of double-positive cells for *PDCD1* and TF in the corresponding TF-positive cells are calculated in the three TIL subsets, respectively.

## Supplementary information

**Additional file 1.** Contains supplemental figures and table outlining the analysis of transcriptome and methylome data from 5 CD8 T cell subtypes of 8 patients.

**Additional file 2.** Review history.

## Data Availability

All the sequencing data were deposited into Genome Sequence Archive database under accession number HRA000059 [57] (https://bigd.big.ac.cn/gsa-human/browse/HRA000059) and GSE141878 (https://www.ncbi.nlm.nih.gov/geo/query/acc.cgi?acc=GSE141878) [58]. ScRNA-Seq data was downloaded from GSE108989 [19] for in silico FACS.
