# Supplementary material for: Distinct epigenetic features of tumor-reactive CD8+ T cells in colorectal cancer patients revealed by genome-wide DNA methylation analysis
Source: Genome Biol. 2019 Dec 31;21:2. doi: 10.1186/s13059-019-1921-y (PMC6937914; doi:10.1186/s13059-019-1921-y)
Supplement: Supplementary file 1 — Additional file 1. Contains supplemental figures and table outlining the analysis of transcriptome and methylome data from 5 CD8 T cell subtypes of 8 patients. [file 13059_2019_1921_MOESM1_ESM.pdf]

Additional File 1
Supplementary Materials

The file includes:

Fig. S1-S8

Table S1

A

Patient ID	Clinical Information				Sequencing methods used	
	Age	Sex	Location	Stage	Smart-seq2	Bisulfite-seq
P0929	58	M	Right colon	IIA	√	√
P1030	58	M	Rectum	IIB	√	√
P1031	79	F	Colon sigmoideum	IVA	√	√
P1101	81	F	Colon transversum	IIIB	√	√
P1112	83	F	Rectum	I	√	√
P1113	83	F	Colon sigmoideum	IIIC	√	√
P1120	74	M	Rectum	I	√	√
P0117	60	F	Right colon	IIIB	√	√

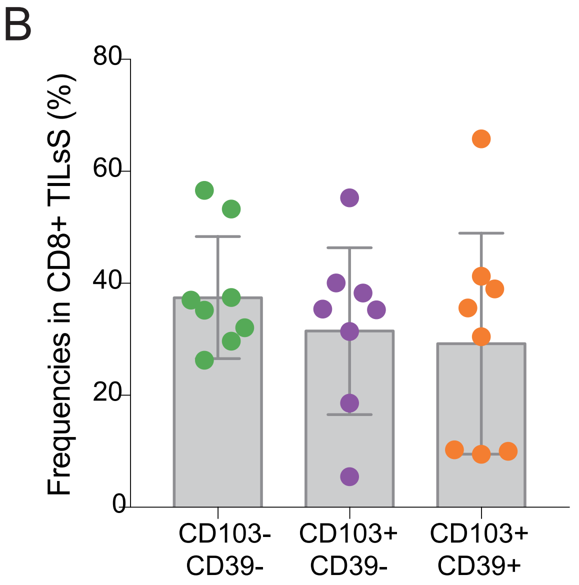

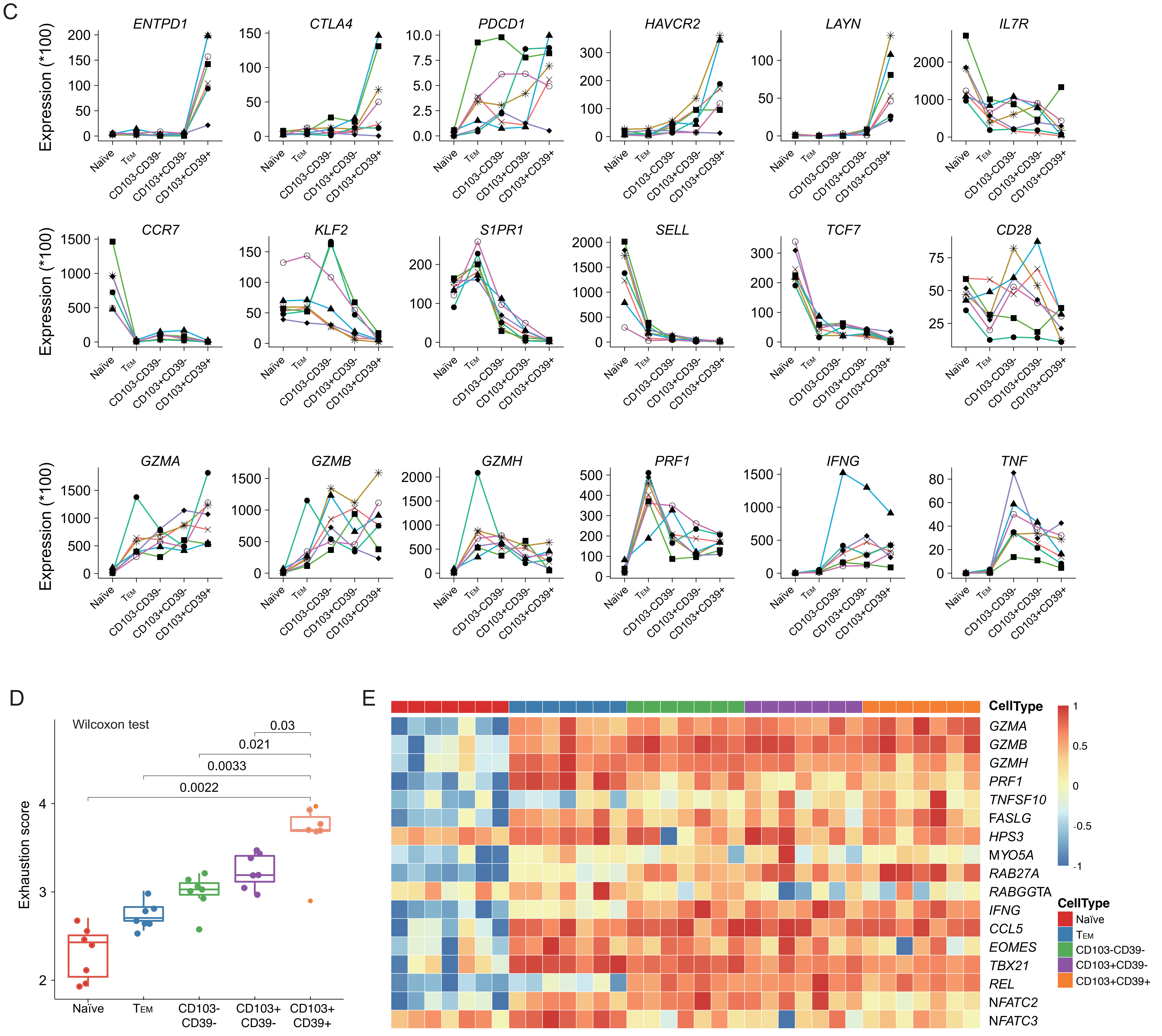

Figure S1. Gene expression analysis revealed a distinct transcriptome of tumor-reactive CD8+ T subtype. (A) The clinical information of CRC patients and sequencing methods used for each patient. (B) Frequencies of Three TIL subtypes in total CD8+ TILs for 8 CRC patients. (C) Examples of immune-related gene expression in five CD8+ T cell populations. Each symbol/line represents one patient, which is connected by a line. (D) Exhaustion levels of five CD8+ T cell populations were analyzed by Wilcoxon test. (E) Heat map displays expression of T_{EM} -related genes in five CD8+ T cell populations.

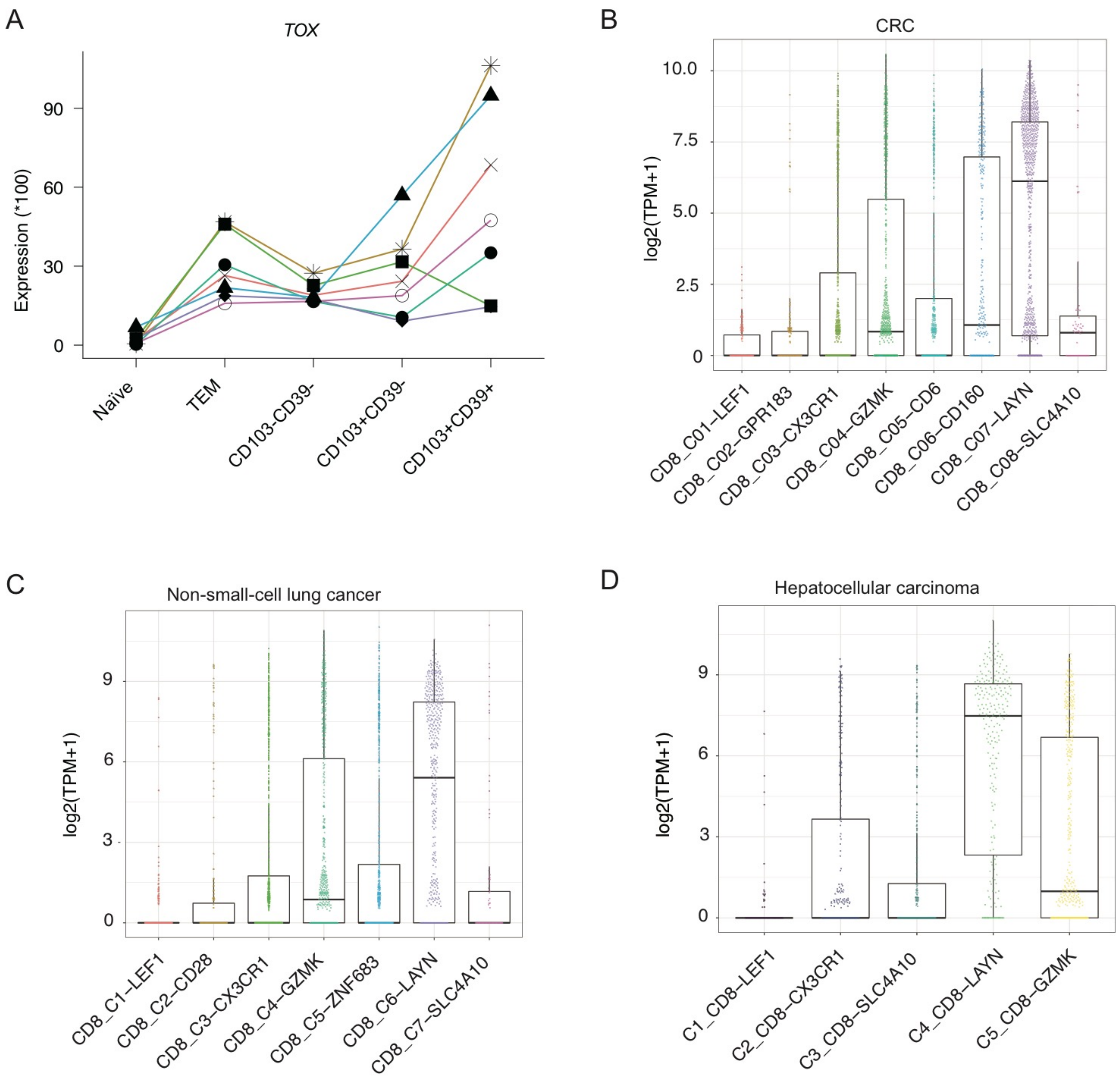

Figure S2. *TOX* is induced in exhausted CD8+ T cells. (A) RNA expression of *TOX* in five subtypes. (B-D) Box plots showing *TOX* expression profiles in different CD8+ T cell clusters from our previous scRNA-seq data of CD8+ T cells in (B) CRC, (C) non-small-cell lung cancer and (D) hepatocellular carcinoma, respectively. Notably, LAYN cluster represents exhausted CD8+ T cells.

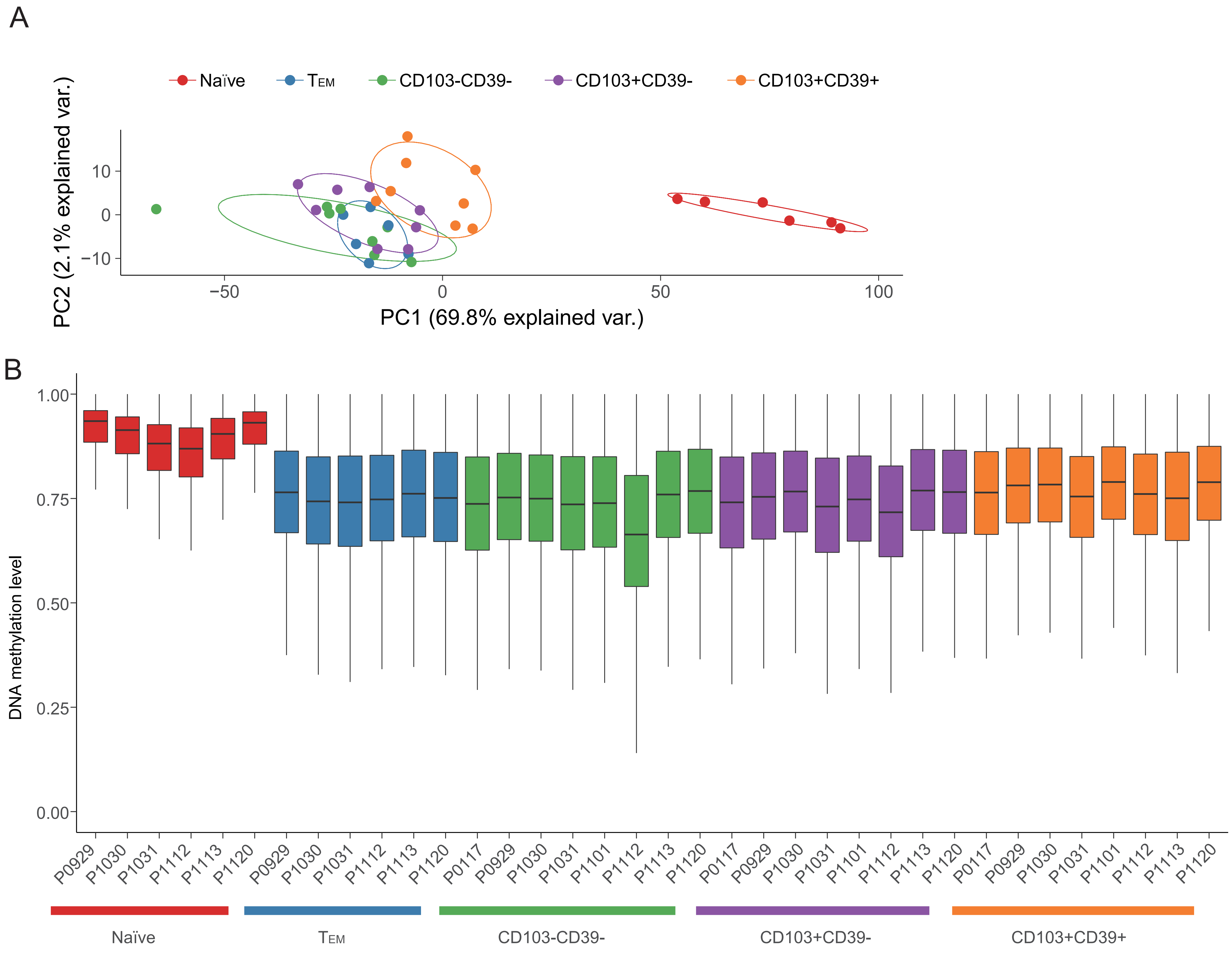

Figure S3. Whole-Genome methylation profiling across multiple CD8+ T cell subtypes. (A) PCA analysis based on methylation profiles of CD8+ T cells in five subtypes. (B) Box plots for the comparison of DNA methylation levels across five subtypes.

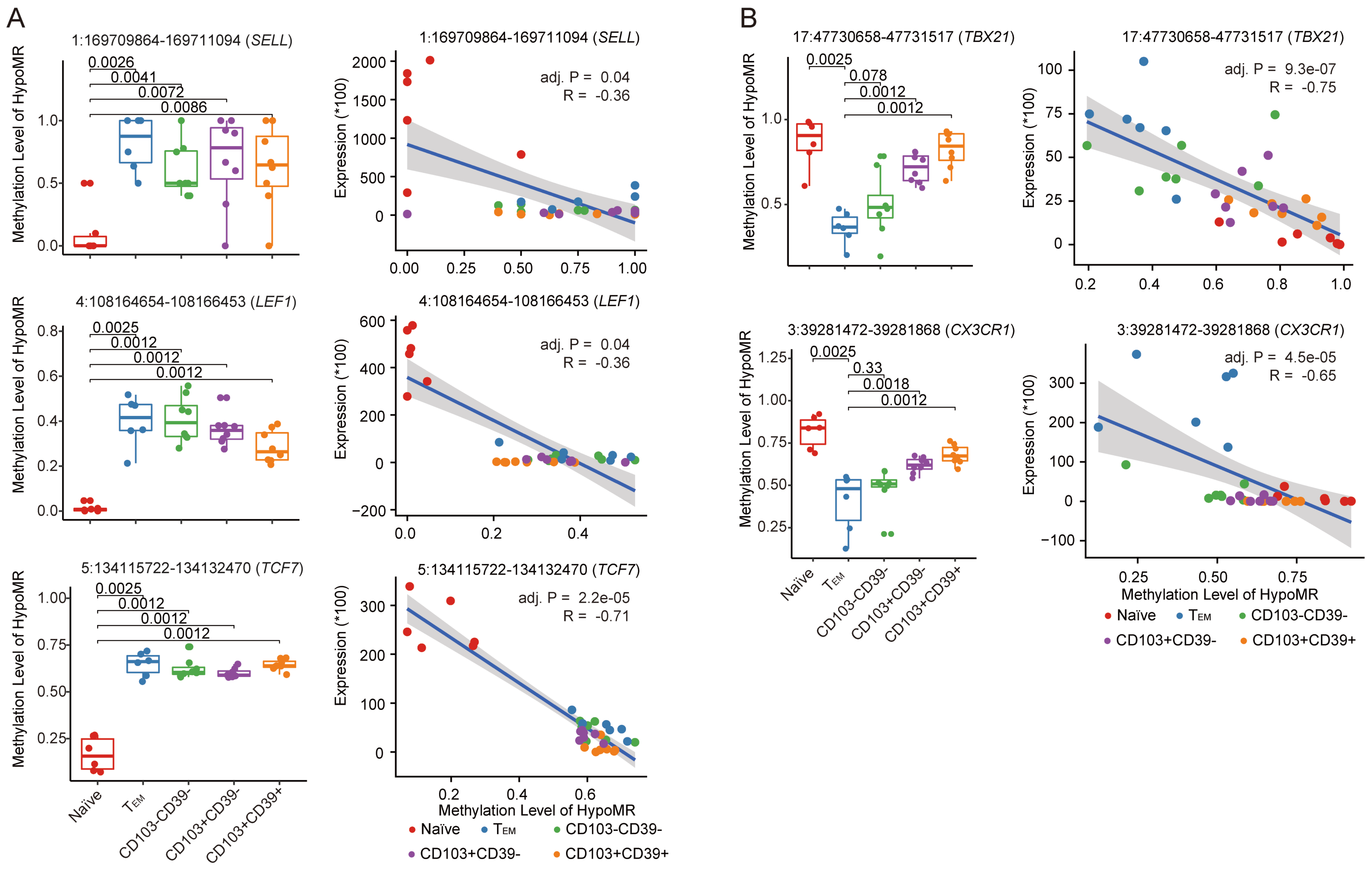

Figure S4. The HypoMR methylation levels of representative genes in naïve and T_{EM} CD8 T cells and their correlations with RNA expression levels across five populations. (A) Representative genes affected by HypoMRs in naïve CD8 T cells. The left panel showed the methylation level of specific HypoMR across the five T cell subtypes. One-tailed wilcoxon test was used to analyze the statistical significance between CD103+CD39+ subtype and other four subtypes. The right panel showed the correlation between RNA expression and HypoMR methylation level. adj.P: Benjamini-Hochberg adjusted p-value. R: regression coefficient. The title of each sub-figure was formatted as “chromosome:start-end(affected gene)”. (B) Representative genes affected by HypoMRs in T_{EM} CD8 T cells. The left and right panels were the same as (A).

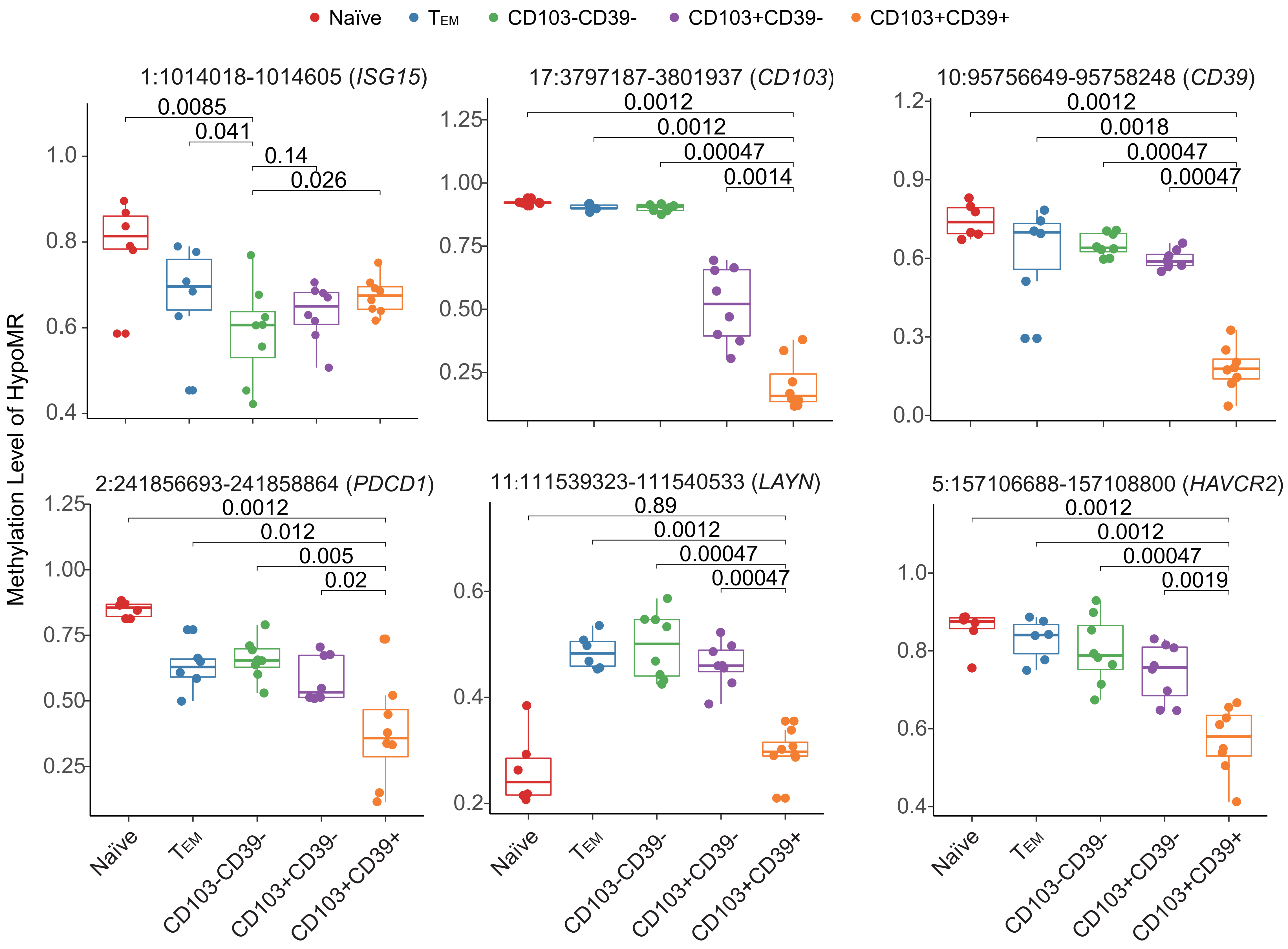

Figure S5. The HypoMR methylation levels of of representative genes across five CD8 T cell populations. One-tailed wilcoxon test was used to analyze the statistical significance between different subtypes. The title of each sub-figure was formatted as "chromosome:start-end(affected gene)".

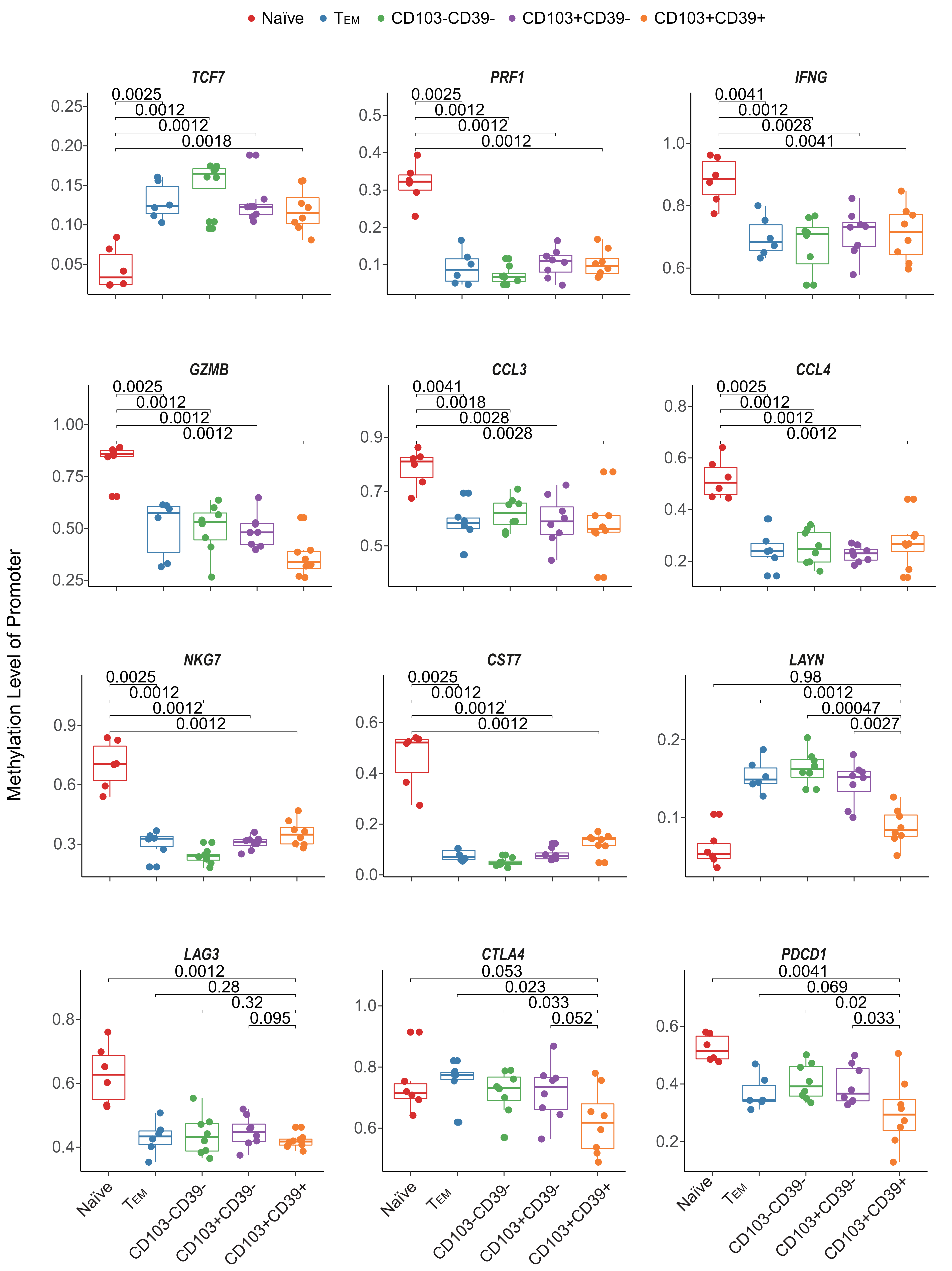

Naïve
TEM
CD103-CD39-
CD103+CD39-
CD103+CD39+
Methylation Level of Promoter
TCF7
0.0025
0.0012
0.0012
0.0018
PRF1
0.0025
0.0012
0.0012
0.0012
IFNG
0.0041
0.0012
0.0028
0.0041
GZMB
0.0025
0.0012
0.0012
0.0012
CCL3
0.0041
0.0018
0.0028
0.0028
CCL4
0.0025
0.0012
0.0012
0.0012
NKG7
0.0025
0.0012
0.0012
0.0012
CST7
0.0025
0.0012
0.0012
0.0012
LAYN
0.98
0.0012
0.00047
0.0027
LAG3
0.0012
0.28
0.32
0.095
CTLA4
0.053
0.023
0.033
0.052
PDCD1
0.0041
0.069
0.02
0.033

Figure S6. The methylation levels of gene promoters across five CD8 T cell subtypes. One-tailed wilcoxon test was used to analyze the statistical significance between different subtypes.

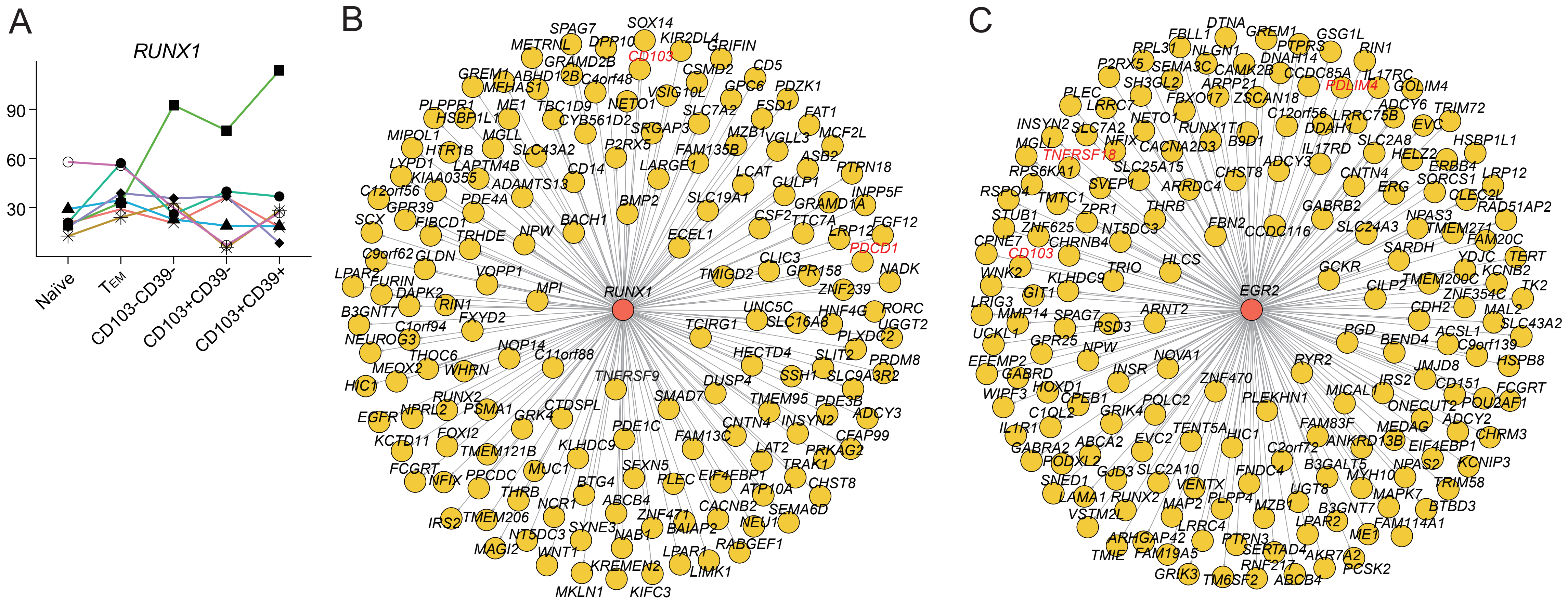

Figure S7. Key transcription factors for CD103+CD39+ subtype. (A) RNA expression of *RUNX1* in five subtypes. (B, C) Predicted targets of (B) *RUNX1* and (C) *EGR2* were displayed. Targets of interest were labeled in red.

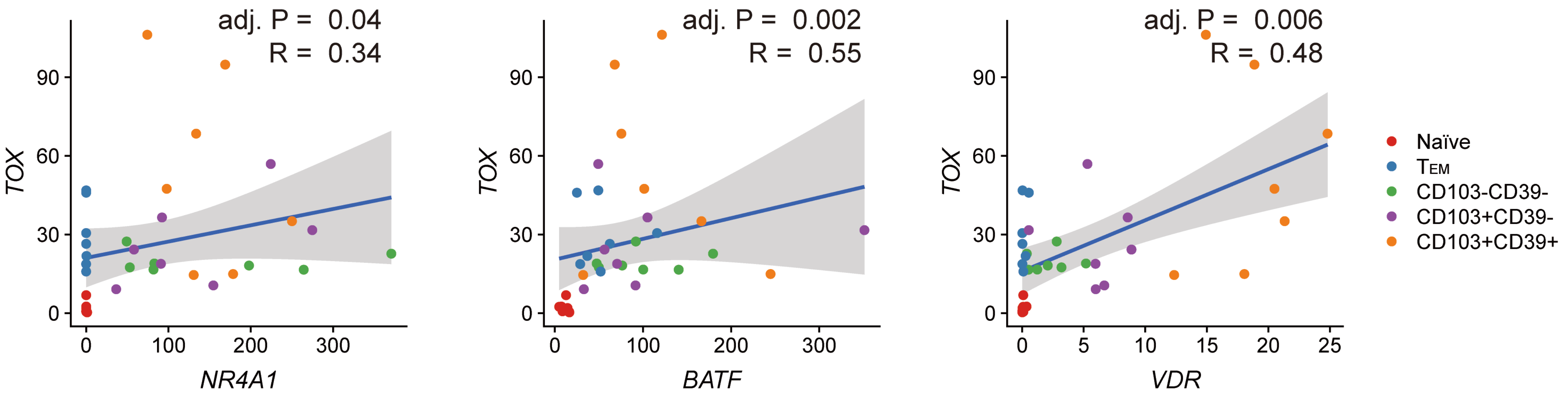

Figure S8. Expression correlation of *TOX* and *NR4A1*, *BATF*, *VDR* in five CD8 T cell populations.

median of covered CpG: 26624899
median of covered CpG by fraction: 0.451475

Naïve T cells
TEM T cells
CD103+CD39+
CD103+CD39-
CD103-CD39-

Patient	Sample	Total_reads	Total_GB	QC_Reads	Mapped_reads	Mapped_ratio	non-CpG_bs	Lambda_bs	Covered_CpGs	Covered_frac
P0929	P1-1_L23_A003	144857804	21.73	143481270	35819145	24.96	0.985187	0.996325	25316945	0.429297
	P1-2_L23_A008	160369528	24.06	158211594	37827232	23.91	0.98311	0.996198	25753434	0.436698
	P2-1_L23_A004	160807964	24.12	158727662	38278395	24.12	0.985238	0.996295	25691277	0.435647
	P2-2_L23_A009	155499252	23.32	151361068	41227040	27.24	0.989246	0.996307	27288506	0.462731
	P3-1_L23_A005	189868220	28.48	187244590	46906145	25.05	0.986103	0.996225	28680269	0.486329
	P3-2_L23_A010	192858134	28.93	190085016	44595736	23.46	0.98534	0.996281	27795898	0.471329
	P4-1_L23_A006	188797732	28.32	185918660	44008803	23.67	0.984573	0.996225	27314056	0.463162
	P4-2_L23_A011	165394086	24.81	161737006	39372608	24.34	0.986713	0.996426	26624899	0.451475
	P5-1_L23_A007	222740972	33.41	219163612	55212634	25.19	0.986483	0.996293	31089377	0.52718
	P5-2_L23_A012	207165724	31.07	203526662	47519057	23.35	0.986125	0.996299	28950498	0.49091
P1030	P1030-1-1-1_HTGW7C	179180718	26.88	174024106	26432757	15.19	0.975764	0.995501	22246378	0.377229
	P1030-1-2-1_HTGW7C	243923314	36.59	234993222	37748251	16.06	0.975639	0.995367	26688835	0.452558
	P1030-1-3-1_HTGW7C	206318860	30.95	197070662	32507057	16.5	0.9757	0.995482	24969435	0.423406
	P1030-1-4-1_HTGW7C	228038518	34.21	219510606	32381554	14.75	0.970754	0.995452	24810411	0.420707
	P1030-1-5-1_HTGW7C	201846596	30.28	192649544	30755469	15.96	0.971685	0.995513	24174702	0.409926
	P1030-2-1-2_HTGW7C	237259734	35.59	223781384	32813064	14.66	0.976926	0.993604	24515052	0.415699
	P1030-2-2-2_HTGW7C	287206650	43.08	271876786	39648695	14.58	0.977548	0.993422	26787026	0.454222
	P1030-2-3-2_HTGW7C	285058550	42.76	264491812	41476278	15.68	0.980722	0.993605	27671885	0.469226
	P1030-2-4-2_HTGW7C	224148840	33.62	205613538	32804166	15.95	0.980194	0.993729	24443574	0.414486
	P1030-2-5-2_HTGW7C	204455744	30.67	180633362	26208704	14.51	0.976197	0.993073	21632656	0.366821
	P1030-3-1-3_HTGW7C	243894334	36.58	235889000	40119826	17.01	0.977359	0.995533	27689832	0.469531
	P1030-3-2-3_HTGW7C	266111622	39.92	256195768	44781917	17.48	0.978338	0.995494	29034828	0.492339
	P1030-3-3-3_HTGW7C	253536002	38.03	244652982	40128840	16.4	0.977528	0.995534	27975490	0.474377
	P1030-3-4-3_HTGW7C	214376246	32.16	205870062	34780240	16.89	0.978685	0.995402	25599763	0.43409
	P1030-3-5-3_HTGW7C	196830880	29.52	183131452	33894297	18.51	0.980547	0.995266	25301285	0.42903
P1031	P1031-1-P1-1_HTGVK(	186765460	28.01	181080936	24554562	13.56	0.978987	0.993377	20658256	0.350301
	P1031-1-P2-1_HTGVK(	222399864	33.36	215757764	30925016	14.33	0.975036	0.993439	23647530	0.40099
	P1031-1-P3-1_HTGVK(	214769116	32.22	207718086	27318414	13.15	0.971912	0.99341	22230004	0.376954
	P1031-1-P4-1_HTGVK(	238155942	35.72	232028242	29761976	12.83	0.969261	0.993491	22979693	0.389665
	P1031-1-P5-1_HTGVK(	198387742	29.76	192542854	27626045	14.35	0.974132	0.993577	22230655	0.376968
	P1031-2-P1-2_HTGW7	255724358	38.36	247849982	37281861	15.04	0.974197	0.994007	26297050	0.445916
	P1031-2-P2-2_HTGW7	215396726	32.31	206860786	33343137	16.12	0.970856	0.994175	25224221	0.427728
	P1031-2-P3-2_HTGW7	219463238	32.92	210620196	32542767	15.45	0.974446	0.994042	24877871	0.421849
	P1031-2-P4-2_HTGW7	166269210	24.94	157369478	23663822	15.04	0.974359	0.994165	20591649	0.349169
	P1031-2-P5-2_HTGW7	219819942	32.97	208637178	32545100	15.6	0.973782	0.99407	24535877	0.416048
	P1031-3-P1-3_HTGVK(	285920448	42.89	275119678	43575495	15.84	0.977364	0.994269	28726383	0.487111
	P1031-3-P2-3_HTGVK(	221728678	33.26	212162626	32888575	15.5	0.977707	0.994259	25175998	0.426904
	P1031-3-P3-3_HTGVK(	234868086	35.23	219986554	33596020	15.27	0.975916	0.994092	25075563	0.425205
	P1031-3-P4-3_HTGVK(	198136754	29.72	187116002	30135799	16.11	0.978597	0.994147	23659312	0.401188
	P1031-3-P5-3_HTGVK(	177753906	26.66	169211880	28466741	16.82	0.979439	0.994206	22850387	0.387474
P1101	P3-1_L4_A008	233050326	34.96	218998162	35386841	16.16	0.987724	0.994789	26508593	0.449509
	P3-2_L3_A010	335491570	50.32	315955066	32221393	10.2	0.979048	0.994939	24529359	0.415946
	P3-3_L2_A009	278435562	41.77	244080018	31521419	12.91	0.974932	0.987718	22217071	0.376735
	P4-1_L4_A009	332620464	49.89	308180738	46639119	15.13	0.98615	0.99483	29885556	0.506767
	P4-2_L3_A011	336890966	50.53	318188084	34689128	10.9	0.980846	0.995197	25804696	0.43757
	P4-3_L2_A011	249155338	37.37	227001400	24749148	10.9	0.972928	0.988586	19325543	0.327704
	P5-1_L4_A010	378938026	56.84	343897778	50139444	14.58	0.985633	0.994945	30683450	0.520289
	P5-2_L3_A012	178567094	26.79	171375332	17934792	10.47	0.984376	0.995371	17796608	0.301779
	P5-3_L2_A012	221684686	33.25	201043102	21755324	10.82	0.969471	0.991765	17658838	0.299442
P1112	1112P1-1_L2_A001	242329132	36.35	241067850	42208985	17.51	0.980843	0.99616	28944875	0.49082
	1112P1-2_L2_A002	272826982	40.92	271286216	49892042	18.39	0.983779	0.996007	30148368	0.511225
	1112P2-1_L2_A004	394821502	59.22	392763952	69909836	17.8	0.984606	0.996024	35274135	0.598141
	1112P2-3_L2_A006	450490368	67.57	448532652	75045663	16.73	0.98093	0.996022	35306024	0.598681
	1112P3-1_L2_A007	293962202	44.09	290997588	43960227	15.11	0.978401	0.995898	22674393	0.384487
	1112P3-2_L2_A008	265061094	39.76	263674088	48997962	18.58	0.976825	0.996262	30833754	0.52285
	1112P4-1_L2_A009	240535982	36.08	239191962	44134368	18.45	0.983778	0.996105	29674868	0.503199
	1112P4-2_L2_A010	358150206	53.72	356333106	63789129	17.9	0.98373	0.9962	33970573	0.57603
	1112P5-1_L2_A011	292194296	43.83	290686054	50875245	17.5	0.985337	0.996218	31442770	0.533175
P1113	1113P1-1_L4_A001	134940296	20.24	134133872	21908545	16.33	0.976897	0.99574	20385280	0.345675
	1113P1-2_L4_A002	395442446	59.32	393072938	62736932	15.96	0.977719	0.995697	32777776	0.555805
	1113P2-1_L4_A004	293259918	43.99	291596862	47267861	16.21	0.979476	0.99572	29586224	0.501694
	1113P2-2_L4_A005	271504624	40.73	270061644	45075982	16.69	0.98055	0.995913	29324470	0.497252
	1113P3-1_L4_A007	265529908	39.83	264083918	46942354	17.78	0.984474	0.996653	30008336	0.508849
	1113P3-2_L4_A008	293202510	43.98	291322458	51260723	17.6	0.982148	0.996525	30451029	0.516359
	1113P4-1_L4_A009	295772280	44.37	294159104	60514368	20.57	0.985819	0.996571	33288340	0.564471
	1113P4-2_L4_A010	444280324	66.64	440645406	76693772	17.4	0.985238	0.996431	35868200	0.608213
	1113P5-1_L4_A011	300581034	45.09	297996062	50636902	16.99	0.982603	0.996554	30754303	0.521501
	1113P5-2_L4_A012	237429208	35.61	234199370	39547488	16.89	0.983056	0.996533	27173305	0.460781

	1113P1-1_L4_A001	134940296	20.24	134133872	21908545	16.33	0.976897	0.99574	20385280	0.345675
	1113P1-2_L4_A002	395442446	59.32	393072938	62736932	15.96	0.977719	0.995697	32777776	0.555805
	1113P2-1_L4_A004	293259918	43.99	291596862	47267861	16.21	0.979476	0.99572	29586224	0.501694
	1113P2-2_L4_A005	271504624	40.73	270061644	45075982	16.69	0.98055	0.995913	29324470	0.497252
P1120	1113P3-1_L4_A007	265529908	39.83	264083918	46942354	17.78	0.984474	0.996653	30008336	0.508849
	1113P3-2_L4_A008	293202510	43.98	291322458	51260723	17.6	0.982148	0.996525	30451029	0.516359
	1113P4-1_L4_A009	295772280	44.37	294159104	60514368	20.57	0.985819	0.996571	33288340	0.564471
	1113P4-2_L4_A010	444280324	66.64	440645406	76693772	17.4	0.985238	0.996431	35868200	0.608213
	1113P5-1_L4_A011	300581034	45.09	297996062	50636902	16.99	0.982603	0.996554	30754303	0.521501
	1113P5-2_L4_A012	237429208	35.61	234199370	39547488	16.89	0.983056	0.996533	27173305	0.460781
	0117P3-1_L2_A004	335771944	50.37	293869202	35255255	12	0.984836	0.995065	25601252	0.434121
	0117P3-2_L2_A005	297634670	44.65	242853254	25966323	10.69	0.984749	0.994775	21528474	0.36506
	0117P3-3_L2_A006	431734528	64.76	345332060	33385454	9.67	0.984306	0.994531	23713703	0.402109
	0117P4-1_L2_A007	324550082	48.68	274836624	32343072	11.77	0.986167	0.994355	24485619	0.415204
P0117	0117P4-2_L2_A008	433115020	64.97	356651126	37400202	10.49	0.984414	0.994867	25932228	0.439726
	0117P4-3_L2_A009	353761964	53.06	306454906	39440724	12.87	0.986059	0.995162	27025106	0.458264
	0117P5-1_L2_A010	423548370	63.53	355336208	39960613	11.25	0.986299	0.994806	26781800	0.454139
	0117P5-2_L2_A011	354669536	53.2	306690600	34974260	11.4	0.985873	0.994601	25735180	0.436387
	0117P5-3_L2_A012	788890	0.12	657704	63440	9.65	0.979232	0.993103	109364	0.00185778

Table S1. Summary of bisulfite-seq data in eight patients.
