# Supplementary material for: Distinct epigenetic features of tumor-reactive CD8+ T cells in colorectal cancer patients revealed by genome-wide DNA methylation analysis
Source: Genome Biol. 2019 Dec 31;21:2. doi: 10.1186/s13059-019-1921-y (PMC6937914; doi:10.1186/s13059-019-1921-y)
Supplement: Supplementary file 2 — Additional file 2. Review history. [file 13059_2019_1921_MOESM2_ESM.docx]

Review History

**First round of review**

**Reviewer 1**

**Are you able to assess all statistics in the manuscript, including the appropriateness of statistical tests used?**

Yes, and I have assessed the statistics in my report.

**Comments to author:**

Yang et al showed that the transcriptomic features of tumor-reactive T cells were shaped by their distinct methylome profile. As expected from the phenotype of tumor reactive CD8 T cells, population markers CD39  and CD103, as well as exhaustion markers PDCD1 , LAYN , and HAVCR2 were specifically demethylated. Authors performed integrated analysis across transcriptome and methylome data combined with binding site enrichment for transcription factors. They identified a novel transcription factor VDR as upregulated in tumor-reactive T cells, and which may regulate exhaustion through PD-1. This has potential to influence the thinking on CD8 T cells in the context of immunotherapy. The use of bystander TILs as a control for tumor-reactive CD8 T cells is a novel and major contribution. However, the paper lacks statistical rigor, and some unwarranted conclusions are drawn from the figures. The manuscript merits reappraisal after a major revision.

General Comments:

1)For cell populations, authors should make the color scheme consistent across all figures and all panels.
2)Adjusted p-values (or q-values) should be used instead of p-values where appropriate.
3) Methods are not sufficiently described, and references to the Methods section are non-existent.

Detailed Comments:

Page 6, line 18: Fig.2C reference is wrong
Page 6, line 21: A more in-depth introduction to TOX, citing recent literature, is warranted. This is given later in the manuscript, but it should be moved up, either here, or to the introduction.
Page 6, line 26-27: Can authors include in Methods a description of how CD3 normalization is performed? If it is TOX - CD3 in the log space, the observed result in Fig S2E may be driven by negative CD3, so that would not be proper normalization. The authors should have a Cox Proportional Hazards model with TOX, CD3, and also tumor type as covariates. What is the significance of TOX in this model? Also, in addition to overall survival, TCGA made available in the final data freeze disease specific survival, progression free interval, and disease free interval as other survival metrics (https://gdc.cancer.gov/about-data/publications/pancanatlas). How does the significance of TOX change across these different metrics, and what may that mean for patients? Also, what do the results look like in colorectal cancer, the focus of the study?
Page 6, line 35-36: It's questionable whether this conclusion (antitumor potential) is supported by the GSVA analysis. Can authors support these results with single genes such as granzyme B and perforin? Fig S1E shows that CD103+CD39+ may not be distinguishable from T_EM cells in terms of antitumor potential.

Page 7, line 15: Figure 2A does not show naive CD8+ T cells. How Figure S3A differs from Figure 2A is not explained in the text, or the legends. Figure S3A should replace Figure 2A, especially since it's difficult to argue there is a statistically significant difference between the CD103-CD39- population and the CD103+CD39- population in Figure 2A.
Page 7, line 16: The authors should clarify whether the TEM  subtype showed a similar or distinct methylation pattern to bystander populations.
Page 7, line 26-27: Are HypoMRs defined in a "one vs rest" fashion, or a pairwise fashion. This is hard to understand from Fig 2B.
Page 7, line 28-29: This conclusion would be warranted by negative correlations in scatter plots of methylation vs expression for LEF1 and SELL.
Page 7, line 30-39: Genes mentioned here should have methylation values visualized with boxplots + statistics to compare across cell populations. Also, CD103 is not shown in Figure 2. TCF7 is shown, also not mentioned in this section.
Page 7, line 43 - Page 8, line 12: Genes mentioned here should have methylation values visualized with boxplots + statistics to compare across cell populations.

Page 9 line 13-24: Can authors include in Methods a detailed description of how the transcriptional networks were generated?

**Reviewer 2**

**Are you able to assess all statistics in the manuscript, including the appropriateness of statistical tests used?**

Yes, and I have assessed the statistics in my report.

**Comments to author:**

CD8+ T cells are important for immune defense, especially for the tumor surveillance. In this manuscript, the authors applied the high throughput transcriptome combined with methylome to study the five major CD8+ T cell subsets from 8 CRC patients to explore the transcriptional regulation signatures of cytotoxic cell, which would be useful with implications for immune therapeutic of cancer.

The authors isolated the tumor-reactive and bystander CD8+ TILs, as well as naïve and effector memory CD8+ T cell subsets as controls from colorectal cancer (CRC) patients. They provided high quality RNA and DNA methylation sequencing data for CRC patients, which would be a valuable resource to the research community.

At the beginning, through the bioinformatics analysis, they found that TOX was expressed highly in the CD103+CD39+ cell types and discovered DNA methylation signatures of genes corresponding to the naïve, cytotoxic and exhausted cells.  In addition, they preformed motif analysis for the hypoMRs for five subsets and tried to find the possible regulators which control the expression of cell type specific genes. At last, they found strong correlation between the transcriptional factors NR4A1, BATF and VDR with the PDCD1 expression and investigated roles of the combination of the TFs and PDCD1 by silico FACS analysis from their previous scRNA-seq. This manuscript is very well written and well organized.

Major:
1.     In the early part of the results, the authors mentioned that TOX was activated in exhausted CD8 T cell from their RNA-seq data, how about the methylation dynamics of TOX in the five subsets?
2.     In Fig.1, the authors preformed the comparison of RNA-seq between the CD103+CD39+ and CD103-CD39-, how about the difference of DNA methylation between different subtypes, especially in these two subtypes?
3.     In the Fig.5, the authors found some transcriptional factors have high correlation with PDCD1, because TOX gene is another key gene for the exhaust of CD8 T cell, are there any important genes correlate with or regulate the expression of the TOX gene?
4.     The authors provide a valuable dataset for the research community. On most occasions, the accession number start from GEO or GSE, please provide the correct GEO number for their dataset.
5.     In eight patients, the proportions of tumor-reactive T cells ranged from to 9.0% to 64.8%, with an average of 29.3%, is there any expression signatures and epigenetic signatures associated with proportions of tumor-reactive T cells?
Minor:

1.     In Fig1.D, E and F, the color for each cell subsets should be consistent.
2.     In Fig2.A, only four subsets were displayed from the PCA figure. However, it seems likely the Fig.S3A containing the PCA of five subsets, what is the difference between the Fig.2A and Fig.S3A?
3.     It would be better if the color of each cell subsets is consistent from the Fig.2A, B and C.
4.     In table S1, please provide detail information about the sample's information, indicating which sample from which cell type?
5.     Fig.2D was not quoted correctly in the manuscript and Fig.2E were not mentioned in the result part.
6.     In Fig.3A, B, C and D, the color for each subset should be consistent.
7.     Please indicate the P-value of the motif analysis for Fig.4A. The figure legend showed that the motifs were from 5 subsets, while only 4 subsets were found in Fig.4A.

**Authors Response**

**Point-by-point responses to the reviewers’ comments:**

Comment

*Response*

Response to Reviewer #1:
Yang et al showed that the transcriptomic features of tumor-reactive T cells were shaped by their distinct methylome profile. As expected from the phenotype of tumor reactive CD8 T cells, population markers CD39 and CD103, as well as exhaustion markers PDCD1, LAYN, and HAVCR2 were specifically demethylated. Authors performed integrated analysis across transcriptome and methylome data combined with binding site enrichment for transcription factors. They identified a novel transcription factor VDR as upregulated in tumor-reactive T cells, and which may regulate exhaustion through PD-1. This has potential to influence the thinking on CD8 T cells in the context of immunotherapy. The use of bystander TILs as a control for tumor-reactive CD8 T cells is a novel and major contribution. However, the paper lacks statistical rigor, and some unwarranted conclusions are drawn from the figures. The manuscript merits reappraisal after a major revision.

*Reply: We thank the reviewer for the overall very positive comments and pointing out the importance of our study that will be of interest to the field of immunotherapy. We also greatly appreciate the detailed comments on “statistical rigor and some unwarranted conclusions”, which have helped us to further improve the overall quality of our manuscript.*

General Comments:
(1) For cell populations, authors should make the color scheme consistent across all figures and all panels.

*Reply: We apologize for the color scheme inconsistency and have made the changes of the color schemes in our revised manuscript.*

(2) Adjusted p-values (or q-values) should be used instead of p-values where appropriate.

*Reply: We appreciate such important comments. As suggested by the reviewer, we have changed to use Benjamini-Hochberg adjusted p-values for all correlation analysis in our manuscript, including both expression-methylation correlation and co-expression analysis.*

(3) Methods are not sufficiently described, and references to the Methods section are non-existent.

*Reply: We apologize that we did not adequately describe our methodology, and have now enhanced our description of Method section and added references to the Methods section where appropriate. All added text was indicated in red color in our revised manuscript.*

Detailed Comments:
Page 6, line 18: Fig.2C reference is wrong

*Reply: Thanks for pointing this out. As suggested, we have made the correction.*

Page 6, line 21: A more in-depth introduction to TOX, citing recent literature, is warranted. This is given later in the manuscript, but it should be moved up, either here, or to the introduction.

*Reply: Thanks for your suggestion, and we agree that we should give an in-depth introduction to TOX earlier. Hence, we have introduced TOX in this part: Recent literatures reported that the thymocyte selection-associated high mobility group box (TOX) protein is required for the development and maintenance of exhausted T cell populations in chronic infection. Removal of its DNA binding domain reduced the expression of PD-1 and resulted in a more polyfunctional T cell phenotype.*

Page 6, line 26-27: Can authors include in Methods a description of how CD3 normalization is performed? If it is TOX - CD3 in the log space, the observed result in Fig S2E may be driven by negative CD3, so that would not be proper normalization. The authors should have a Cox Proportional Hazards model with TOX, CD3, and also tumor type as covariates. What is the significance of TOX in this model? Also, in addition to overall survival, TCGA made available in the final data freeze disease specific survival, progression free interval, and disease-free interval as other survival metrics (https://gdc.cancer.gov/about-data/publications/pancanatlas). How does the significance of TOX change across these different metrics, and what may that mean for patients? Also, what do the results look like in colorectal cancer, the focus of the study?

*Reply: We appreciate the insightful comments by the reviewer. Survival analysis was conducted by GEPIA2, a powerful web server provided by our lab, which intergates both log-rank test and Cox proportional hazard model (Tang et al., 2019). In addition to overall survival, GEPIA2 also intergrates disease free survival, another widely-used metric for survival analysis.
To answer your question, CD3 normalization means using TOX/CD3D relative expression levels as the input for survival analysis to remove the effect of the difference in T cell infiltrating.
Besides, We realized that it was unreasonable and inexplicable to put samples from different tumors together. Therefore, we investigated the relationship of TOX expression and patients’ survival time across 33 tumor types independently by using the Survival Map function of GEPIA2. However, this correlation is not consistent within every tumor type (Fig. R1A). For example, the increase of TOX expression is at higher risk in KIRP, while is at lower risk in BRCA (Fig. R1A). More importantly, for the survival analysis of CRC patients (which combines COAD and READ in TCGA), we didn’t observe significant correlation of TOX expression and overall survival (p=0.86) or disease-free survival (p=0.07) in both log-rank test and Cox proportional hazard model (Fig. R1B, C). Overall, these results suggest that the relationship between TOX expression and survival is more complex than we thought. In order to increase the rigour of our manuscript, we decided to remove Fig S2E. We thank the reviewer for pointing out this and helping us improve our manuscript.*


*Fig. R1 The associations of TOX expression and survival rates. (A) The overall survival map and disease-free survival map across different tumor types. The heat map shows the hazard ratios in logarithmic scale (log10) for TOX. The red and blue blocks denote higher and lower risks, respectively. The rectangles with frames mean the significant results (p < 0.05). (B, C) The Overall survival rate (B) and disease-free survival rate (C) from TCGA data of CRC patients.*

Page 6, line 35-36: It's questionable whether this conclusion (antitumor potential) is supported by the GSVA analysis. Can authors support these results with single genes such as granzyme B and perforin? Fig S1E shows that CD103+CD39+ may not be distinguishable from T_EM cells in terms of antitumor potential.

*Reply: Following the helpful suggestions by the reviewer, we have investigated the expression of several genes associated with antitumor potential and found that high gene expression level of granzyme A/B/H, PRF1, IFNG and TNF was observed in CD39+CD103+ subset. To make this point clearer, we have now extended the descriptions in our main text as “Furthermore, we analyzed effector function of these CD8 T cell subtypes by the expression of granzyme A/B/H, cytotoxic granules PRF1, interferon (IFN)-γ and tumor necrosis factor (TNF). Interesting, we found that exhausted CD103+CD39+ subtype still had relatively high expression of these cytotoxic proteins (Fig. S1C). Together with the GSVA results, it indicates that CD103+CD39+ subtype may not have lost their antitumor potential.”*

Page 7, line 15: Figure 2A does not show naive CD8+ T cells. How Figure S3A differs from Figure 2A is not explained in the text, or the legends. Figure S3A should replace Figure 2A, especially since it's difficult to argue there is a statistically significant difference between the CD103-CD39- population and the CD103+CD39- population in Figure 2A.

*Reply: We apologize for the confusion. Naïve T cells is a very distinct subtype, so we only showed the PCA results of the rest four subtypes in Fig. 2A to give a better visualization. We have corrected the legends of Fig. 2A.*

Page 7, line 16: The authors should clarify whether the TEM subtype showed a similar or distinct methylation pattern to bystander populations.

*Reply: Thanks for your comment. Our previous conclusion is indeed inaccurate. We have modified the sentence to “The TEM subtype showed a similar methylation pattern to both bystander populations”.*

Page 7, line 26-27: Are HypoMRs defined in a "one vs rest" fashion, or a pairwise fashion. This is hard to understand from Fig 2B.

*Reply: We apologize that we did not adequately explain how HypoMRs were defined in methods, which can be very confusing. To make it clear, we have changed the sentence in the main text to: “We calculated hypomethylated regions (HypoMRs) for each T cell population in a "one vs rest" fashion” and revised the sentence in Method section as “Specific hypomethylated regions (HypoMRs) for each T cell population were calculated in a "one vs rest" fashion by using radmeth regression, radmeth adjust and radmeth merge commands in MethPipe”.*

Page 7, line 28-29: This conclusion would be warranted by negative correlations in scatter plots of methylation vs expression for LEF1 and SELL.

*Reply: This is an excellent point and we thank the reviewer for providing specific suggestion about the negative correlation to support the conlusion of “their enhanced gene expression”. Following this helpful suggestion, we have performed the correlation analysis of gene expression and DNA methylation and found that significantly negative correlations were observed for LEF1, SELL, TBX21 and CX3CR1. Hence, to support the conclusion, we have added a new figure (Fig. S4) into the main text. We thank the reviewer for helping us improve our manuscript.*

Page 7, line 30-39: Genes mentioned here should have methylation values visualized with boxplots + statistics to compare across cell populations. Also, CD103 is not shown in Figure2. TCF7 is shown, also not mentioned in this section.

*Reply: We thank the reviewer for this suggestion, and we have added two new figures of boxplots to compare the methylation levels across cell populations (Fig. S4; Fig. S5). Also, we corrected the mistakes for CD103 and TCF7.*

Page 7, line 43 - Page 8, line 12: Genes mentioned here should have methylation values visualized with boxplots + statistics to compare across cell populations.

*Reply: We thank the reviewer for this suggestion, and we have revised it in Fig. S6.*

Page 9 line 13-24: Can authors include in Methods a detailed description of how the transcriptional networks were generated?

*Reply: We apologize that we did not adequately describe it in the manuscript. To make it clearer, we have added a new paragraph to describe the process of “Inferred regulation network construction” in the Method section as followed: “First, the enriched transcription factor motifs within HypoMRs were performed by HOMER to identify possible key transcription factors in each T cell population. Furthermore, to investigate the possible target genes regulated by transcription factors in tumor-reactive T cells, transcription factor binding motifs were scanned through the promoter regions in the whole human genome (using scanMotifGenomeWide.pl in homer) to identify all possible target sites geome-wide, and then all the predicted target sites were intersected with the specific hypomethylated regions in tumor-reactive T cell population to construct the possible regulation network in each T cell population.”*

Response to Reviewer #2:
CD8+ T cells are important for immune defense, especially for the tumor surveillance. In this manuscript, the authors applied the high throughput transcriptome combined with methylome to study the five major CD8+ T cell subsets from 8 CRC patients to explore the transcriptional regulation signatures of cytotoxic cell, which would be useful with implications for immune therapeutic of cancer.

The authors isolated the tumor-reactive and bystander CD8+ TILs, as well as naïve and effector memory CD8+ T cell subsets as controls from colorectal cancer (CRC) patients. They provided high quality RNA and DNA methylation sequencing data for CRC patients, which would be a valuable resource to the research community.

At the beginning, through the bioinformatics analysis, they found that TOX was expressed highly in the CD103+CD39+ cell types and discovered DNA methylation signatures of genes corresponding to the naïve, cytotoxic and exhausted cells. In addition, they performed motif analysis for the hypoMRs for five subsets and tried to find the possible regulators which control the expression of cell type specific genes. At last, they found strong correlation between the transcriptional factors NR4A1, BATF and VDR with the PDCD1 expression and investigated roles of the combination of the TFs and PDCD1 by silico FACS analysis from their previous scRNA-seq. This manuscript is very well written and well organized.

*Reply: We thank the reviewer for the overall enthusiastic comments and greatly appreciate all the constructive comments and suggestions.*

Major:
1. In the early part of the results, the authors mentioned that TOX was activated in exhausted CD8 T cell from their RNA-seq data, how about the methylation dynamics of TOX in the five subsets?

*Reply: We thank the reviewer for the interesting question about the methylation dynamics of TOX. In fact, we have calculated the promoter methylation level of TOX, and found that the methylation level of TOX was consistently low and without any significant difference in all the five subsets (Fig. R2). Therefore, we suspect that the activation of TOX may not only depend on DNA methylation, but also some other factors.*

*Fig. R2 Methylation level of TOX promoter in five CD8 T cell subtypes. Wilcoxon test was used to analyze the statistical significance between CD103+CD39+ subtype and other four subtypes.*

2. In Fig.1, the authors preformed the comparison of RNA-seq between the CD103+CD39+ and CD103-CD39-, how about the difference of DNA methylation between different subtypes, especially in these two subtypes?

*Reply: We apologize that we did not adequately explain how hypomethylated regions (HypoMRs) were defined, which led to your misunderstanding. To make it clear, we have changed the sentence in the main text to: “We calculated hypomethylated regions (HypoMRs) for each T cell population in a "one vs rest" fashion” and revised the sentence in Method accordingly. Specific HypoMRs for each T cell population were displayed in Fig. 2C and selected genes associated with the HypoMRs in each subset were list at the left side of the figure. Particularly, compared with other T substypes, CD103+CD39+ T cells showed demethylation of the markers for tumor reactivity (CD39 and CD103) and exhaustion markers (PDCD1, HAVCR2, and LAYN) (Fig. 2C; Fig. S5).*

3. In the Fig.5, the authors found some transcriptional factors have high correlation with PDCD1, because TOX gene is another key gene for the exhaust of CD8 T cell, are there any important genes correlate with or regulate the expression of the TOX gene?

*Reply: We thank the reviewer for the interesting question about the expression regulation of TOX. Due to our limited sample size, here we could only focus on the expression correlation between TOX and the three transcription factors we mentioned (NR4A1, BATF and VDR). Interestingly, we found that VDR and TOX had a significant expression correlation (Fig. R4), which further supports a role of VDR in regulating T cell exhaustion. We would like to explore more in the expression regulation of TOX by VDR, such as by ChIP-seq.

Fig. R4 Expression correlation of TOX and NR4A1, BATF, VDR in five CD8 T cell populations.*

4. The authors provide a valuable dataset for the research community. On most occasions, the accession number start from GEO or GSE, please provide the correct GEO number for their dataset.

*Reply: We thank the reviewer for pointing out the importance of our dataset and we have uploaded our data into GSA (Genome Sequence Archive) under accession number PRJCA001585. The data would be accessible upon the acceptance of the paper.*

5. In eight patients, the proportions of tumor-reactive T cells ranged from to 9.0% to 64.8%, with an average of 29.3%, is there any expression signatures and epigenetic signatures associated with proportions of tumor-reactive T cells?

*Reply: We thank the reviewer for the insightful comment. However, there is no report that any expression signatures and epigenetic signatures are associated with the proportions of tumor-reactive T cells. According to previous research, the proportions of tumor-reactive T cells in colorectal cancers are correlated with mutation burden levels (Duhen et al., 2018). CD8 TILs from patients with microsatellite instability-high (MSIhigh) colon cancer are reported to have a high frequency of tumor-reactive T cells (Duhen et al., 2018). Duet to the limited sample size and only one MSI patient in our study, we could not investigate their relationship with frequency of tumor-reactive T cells.

The molecular mechanism that determines the proportion of tumor-reactive T cells would have very important clinical meanings, such as in immunotherapy. In our revised manuscript, we have added sentences into the Discussion section as “In addition, it is intriguing to find out the molecular mechanism determining the various proportions of tumor-reactive T cells in different patients, which can have important clinical meanings. For instance, patients whose tumors had a higher percentage of tumor-reactive CD8 TILs at the time of surgery correlated with better overall survival (3). Duhen et al. showed that the highest percentage of tumor-reactive CD8 TILs were found in melanoma and microsatellite instability (MSI)high colon cancer, both tumors with high mutational burden (3). Other molecular mechanism such as epigenetic regulation associated with proportions of tumor-reactive T cells remains to be explored in the future.”*

Minor:
1. In Fig1.D, E and F, the color for each cell subsets should be consistent.

*Reply: Thanks for pointing this out. We have made the changes in our revised manuscript.*

2. In Fig. 2A, only four subsets were displayed from the PCA figure. However, it seems likely the Fig.S3A containing the PCA of five subsets, what is the difference between the Fig.2A and Fig.S3A?

*Reply: We apologize for the confusion. Naïve T cells is a very distinct subtype, so we only showed the PCA results of the rest four subtypes in Fig. 2A to give a better visualization. We have corrected the legends of Fig. 2A.*

3. It would be better if the color of each cell subsets is consistent from the Fig.2A, B and C.

*Reply: As suggested by the reviewer, we have made the corresponding changes.*

4. In table S1, please provide detail information about the sample's information, indicating which sample from which cell type?

*Reply: Thanks for your advice, we have added cell type information in Table S1.*

5. Fig.2D was not quoted correctly in the manuscript and Fig.2D were not mentioned in the result part.

*Reply: (1) Sorry for this mistake, we have corrected it. (2) Fig. 2D is mentioned in the results of revised manuscript.*

6. In Fig.3A, B, C and D, the color for each subset should be consistent.

*Reply: As suggested by the reviewer, we have made the corresponding changes.*

7. Please indicate the P-value of the motif analysis for Fig.4A. The figure legend showed that the motifs were from 5 subsets, while only 4 subsets were found in Fig.4A.

*Reply: Thanks for pointing this out. (1) In our revised manuscript, we added the adjusted p-values (q-values) of motif analysis in Fig.4A. (2) We combined TEM and CD103-CD39- subsets as they have the same enriched TFs in the table.


References
Duhen, T., Duhen, R., Montler, R., Moses, J., Moudgil, T., de Miranda, N.F., Goodall, C.P., Blair, T.C., Fox, B.A., McDermott, J.E., et al. (2018). Co-expression of CD39 and CD103 identifies tumor-reactive CD8 T cells in human solid tumors. Nat Commun 9, 2724.
Tang, Z., Kang,We are grateful to reviewers for their insightful and constructive comments and suggestions, which are very helpful to improve and strengthen our manuscript. Point-by-point responses are presented below. (The reviewer’s comments are in italics)*

**Second round of review**

**Reviewer 1**

The authors have satisfactorily responded to my review, and significantly improved the manuscript. I am happy to recommend the publication of this manuscript.

**Reviewer 2**

The authors have answered all the questions I raised.

The only concern is that in the end of results part, the author mentioned Fig. S8A in the main text, but this figure seems not included in their supplementary data.

**Authors Response**

**Point-by-point responses to the reviewers’ comments:**

The only concern is that in the end of results part, the author mentioned Fig. S8A in the main text, but this figure seems not included in their supplementary data.

*We have added Fig. S8 in the revised manuscript, thanks for pointing this out.*
